# The Global Retinoblastoma Outcome Study: a prospective, cluster-based analysis of 4064 patients from 149 countries

**DOI:** 10.1016/S2214-109X(22)00250-9

**Published:** 2022-08

**Authors:** The Global Retinoblastoma Study Group

## Abstract

**Background:**

Retinoblastoma is the most common intraocular cancer worldwide. There is some evidence to suggest that major differences exist in treatment outcomes for children with retinoblastoma from different regions, but these differences have not been assessed on a global scale. We aimed to report 3-year outcomes for children with retinoblastoma globally and to investigate factors associated with survival.

**Methods:**

We did a prospective cluster-based analysis of treatment-naive patients with retinoblastoma who were diagnosed between Jan 1, 2017, and Dec 31, 2017, then treated and followed up for 3 years. Patients were recruited from 260 specialised treatment centres worldwide. Data were obtained from participating centres on primary and additional treatments, duration of follow-up, metastasis, eye globe salvage, and survival outcome. We analysed time to death and time to enucleation with Cox regression models.

**Findings:**

The cohort included 4064 children from 149 countries. The median age at diagnosis was 23·2 months (IQR 11·0–36·5). Extraocular tumour spread (cT4 of the cTNMH classification) at diagnosis was reported in five (0·8%) of 636 children from high-income countries, 55 (5·4%) of 1027 children from upper-middle-income countries, 342 (19·7%) of 1738 children from lower-middle-income countries, and 196 (42·9%) of 457 children from low-income countries. Enucleation surgery was available for all children and intravenous chemotherapy was available for 4014 (98·8%) of 4064 children. The 3-year survival rate was 99·5% (95% CI 98·8–100·0) for children from high-income countries, 91·2% (89·5–93·0) for children from upper-middle-income countries, 80·3% (78·3–82·3) for children from lower-middle-income countries, and 57·3% (52·1-63·0) for children from low-income countries. On analysis, independent factors for worse survival were residence in low-income countries compared to high-income countries (hazard ratio 16·67; 95% CI 4·76–50·00), cT4 advanced tumour compared to cT1 (8·98; 4·44–18·18), and older age at diagnosis in children up to 3 years (1·38 per year; 1·23–1·56). For children aged 3–7 years, the mortality risk decreased slightly (p=0·0104 for the change in slope).

**Interpretation:**

This study, estimated to include approximately half of all new retinoblastoma cases worldwide in 2017, shows profound inequity in survival of children depending on the national income level of their country of residence. In high-income countries, death from retinoblastoma is rare, whereas in low-income countries estimated 3-year survival is just over 50%. Although essential treatments are available in nearly all countries, early diagnosis and treatment in low-income countries are key to improving survival outcomes.

**Funding:**

Queen Elizabeth Diamond Jubilee Trust.

## Introduction

Retinoblastoma is the most common cause of death from eye cancer worldwide.^1^ Early diagnosis and prompt treatment can save a child’s life and the eye globe, with useful vision retained in selected cases.

Being a rare malignancy, data on retinoblastoma outcomes are sparse, especially from low-income and middle-income countries.^2^ Most evidence is from treatment centres in high-income countries, although patients in high-income countries of North America, Europe, and Oceania combined represent less than 10% of global cases, whereas more than 80% of patients with retinoblastoma reside in low-income and middle-income countries in Africa, Asia, and Latin America.^3^

In a single-centre study done in the USA (1994–2014),^4^ the retinoblastoma survival rate was reported to reach 99% (mean follow-up 4–8 years, depending on age group); in a single-centre study done in the UK (2002–14),^5^ it was reported to reach 100% (mean follow-up 5 years); and in a single-centre study done in Japan (1984–2016),^6^ it was reported to reach 95% (10-year overall survival), with most deaths occurring from trilateral retinoblastoma in all three studies. In low-income countries, survival rates are significantly lower: 60% (5-year survival) in a single-centre study done in Uganda (2009–19),^7^ 53% (follow-up time not indicated) in a single-centre study done in Senegal (2006–10),^8^ and 24% (10-year survival) in a single-centre study done in Nepal (1998–2008),^9^ with the majority of deaths in all three studies occurring from metastatic spread.

We previously reported the clinical characteristics of a global sample of children with retinoblastoma at the time of diagnosis, who presented to specialised treatment centres across the world in 2017.^3^ Late presentation with advanced disease was strongly associated with the economic grouping of the country of residence. Here, we aimed to investigate 3-year outcomes in the same global cohort of children with retinoblastoma.

## Methods

### Study design and participants

This study adhered to the Guidelines for Accurate and Transparent Health Estimates Reporting (GATHER) statement^10^ and the STrengthening the Reporting of OBservational studies in Epidemiology (STROBE) statement.^11^ This prospective cluster-based analysis followed up the cohort of 4351 children with retinoblastoma from 153 countries who presented to 278 treatment centres worldwide in 2017 (known as the Global Retinoblastoma Presentation Study).^3^ Details of the Global Retinoblastoma Presentation Study have been reported previously.^3^ In brief, during 2017–18, all known retinoblastoma centres across the world were contacted to form a global network. Centres involved in the diagnosis and treatment of patients with retinoblastoma were eligible to participate. The Global Retinoblastoma Presentation Study was a 1-year cross-sectional analysis that included all treatment-naive patients with retinoblastoma who presented to participating centres from Jan 1, 2017, to Dec 31, 2017, and who were treated or offered treatment for retinoblastoma. Following the Global Retinoblastoma Presentation study, which focused on clinical and epidemiological data at the time of diagnosis, all centres were invited to participate in a prospective analysis to report 3-year outcomes of patients from the original sample.

Participating centres were asked to submit information about primary and additional treatments, duration of follow-up, metastasis, eye globe salvage, and survival outcome. Information about the impact of the COVID-19 pandemic was also requested, as the pandemic emerged during the study period. All data were combined with the previously reported presentation data,^3^ including sex, age at diagnosis, laterality, familial retinoblastoma, and clinical tumour, node, metastasis, and heredity (cTNMH) stage.^12^

Using the Global Retinoblastoma Study Group network, we also attempted to contact additional treatment centres that had not previously participated in the Global Retinoblastoma Presentation Study. New or existing centres that added new patients were asked to submit both the presentation data and the outcome data. All participating centres were asked to submit the completed forms in early 2020; however, because of the COVID-19 pandemic, the first form was received on July 3, 2020, and the last on March 31, 2021. For each form received, a process of data quality assurance was done, as previously described.^3^ Information on sex, the patient’s country of residence, laterality, and death status at last follow-up (including unknown) was obligatory for a patient to be included.

This study was approved by the Institutional Review Board of the London School of Hygiene & Tropical Medicine (London, UK), which granted a waiver of informed consent. Participating centres applied for and received local ethics approval.

### Data analysis

Analyses were done with R software (version 3.5.2). For survival analysis, all-cause mortality was used. Time from diagnosis to death was summarised by Kaplan-Meier curves. The analysis of time from diagnosis to enucleation (or exenteration) accounted for the competing risk of death, as patients who die with an intact eye globe present a special type of censoring. We used the standard approach of computing cumulative incidence curves for enucleation, adjusted for the competing risk of death.^13^ These curves provide consistent estimates of the proportion of patients who will undergo enucleation by a given time, accounting for prior death. In the case of bilateral eye globe loss, the first event was used for analysis.

For both time to death and time to enucleation, the association with potential risk factors or protective factors was analysed with Cox regression models. Smoothing splines were fitted for continuous factors to assess possible non-linear associations with risk. When such associations were found, they were replaced by linear splines for ease of interpretation. The knot locations for the linear splines were fixed on the basis of the smoothing spline analysis. The proportionality assumption of the Cox regression model was checked with Schoenfeld residuals.

All models included as potential risk or protective factors the economic grouping of the patient’s country,^14^ primary tumour stage (cT) and hereditary (H) category according to the eighth edition of the American Joint Committee on Cancer (AJCC) Staging Manual,^12^ age at diagnosis, and indicators for sex, laterality, and familial retinoblastoma. Age at diagnosis was found to have a non-linear relationship with both survival and eye globe salvage. The preliminary analyses were done with smoothing splines, and the subsequent analyses with linear splines are shown in the appendix (pp 1–2). All analyses were clustered by treatment centre nested within the patient’s country of residence. Robust standard errors, reflecting the clustering, were used to compute two-tailed p values and 95% CIs. Initial p values are presented, also after correction with Bonferroni’s method (multiplied by 13, the number of terms in each Cox model).

Missing values in the risk factors and protective factors were imputed as the most common value (for categorical variables) or the median value (for age at diagnosis) within the patient’s economic group. To account for missing outcome data, the analyses used inverse probability weighting, assuming data were missing at random.^15^ The probability of having data was estimated separately for each outcome by fitting a logistic regression model using the same factors listed above as predictors. Linear relationships to all factors were assumed in these models. Missing data for any of the categorical variables defined an additional category, so that the model accounted for the fact that patients with any of these missing data might also be more likely to have missing data on outcomes. Missing age at diagnosis was handled by assigning the average age in place of the missing values for age, and including an indicator for those missing the predictor. Patients declared to be alive or have an unknown death status and a follow-up time of zero were treated as missing the outcome.

Sensitivity analyses were done to ascertain whether the above decisions had an effect on the findings. These analyses included the use of inverse probability weights, imputation of missing factors versus deletion, the simple imputation versus multiple imputation from marginal distributions, extending the declaration of missing survival outcomes to participants with a follow-up of 1 month or less, and the possibility that the relationship of survival or eye globe salvage to age at diagnosis differs by hereditary status.

### Role of the funding source

The funder had no role in study design, data collection, data analysis, data interpretation, or writing of the report.

## Results

**Figure 1:**
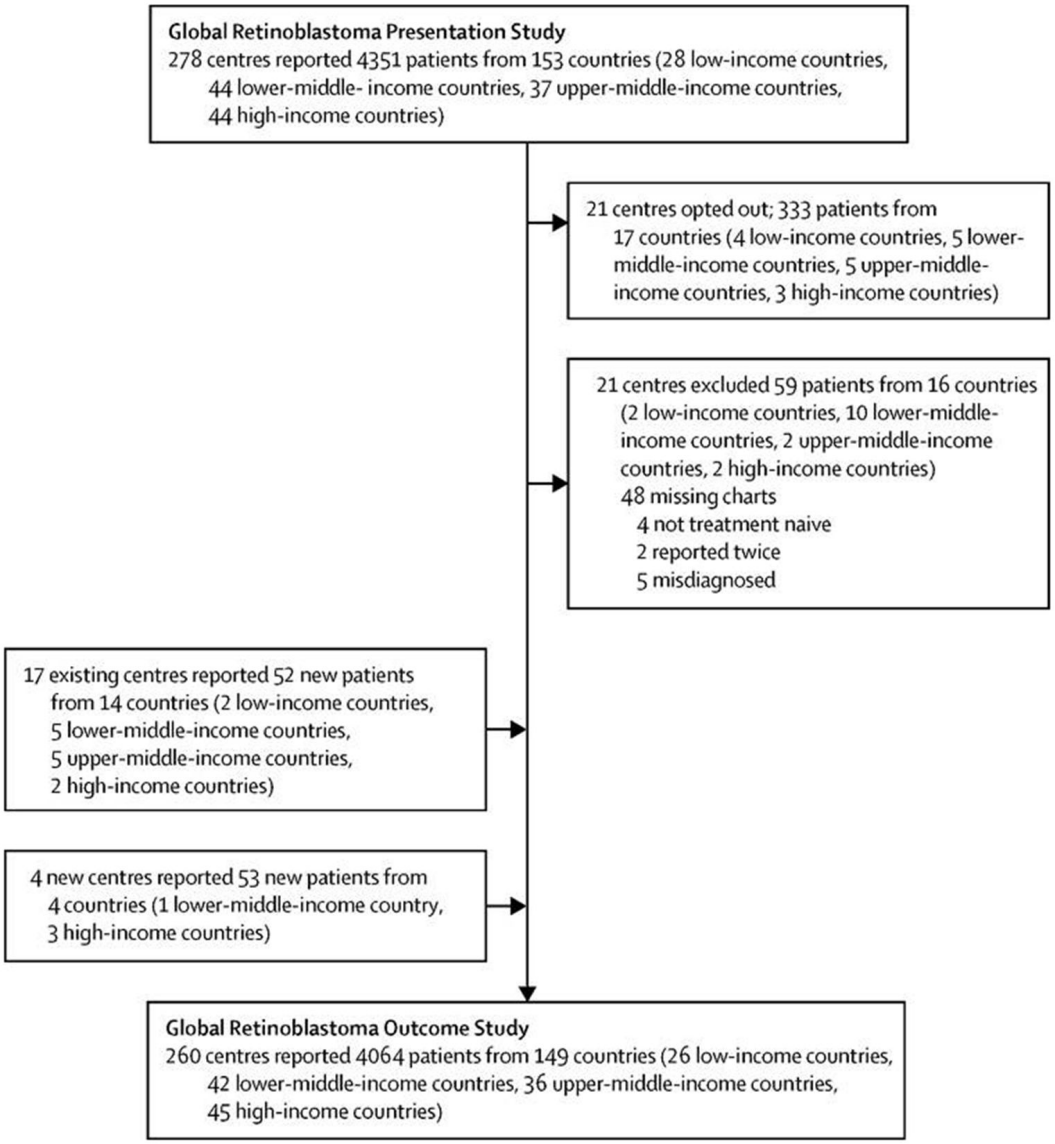
Study flowchart Of the original 278 centres that participated in the Global Retinoblastoma Presentation Study, 21 opted out of the Global Retinoblastoma Outcome Study, a single centre from an African country had originally reported data for a single child, which was later excluded because of misdiagnosis, and four new centres joined the Global Retinoblastoma Outcome Study, reaching a total of 260 participating centres.

Of the 4351 patients included in the Global Retinoblastoma Presentation Study, 59 were excluded from the present analysis because of unavailable data, misdiagnosis, unmet inclusion criteria, or duplicate reporting, and 333 opted out as the corresponding centres did not want to participate in the study. An additional 105 newly recruited patients were added to the cohort. Overall, the study cohort therefore comprised 4064 treatment-naive patients, residing in 149 countries, who presented to 260 treatment centres worldwide in 2017, and received or were offered treatment for retinoblastoma (figure 1). 480 (11·8%) of 4064 children were from low-income countries, 1791 (44·1%) were from lower-middle-income countries, 1151 (28·3%) were from upper-middle-income countries, and 642 (15·8%) were from high-income countries. Asia had 2112 (52·0%) of 4064 cases, Africa had 958 (23·6%), Europe had 483 (11·9%), Latin America and the Caribbean had 309 (7·6%), North America had 182 (4·5%), and Oceania had 20 (0·5%).

The median age at diagnosis was 23·2 months (IQR 11·0–36·5), 1827 (45·0%) of 4064 patients were girls, 2809 (69·1%) of 4064 presented with unilateral disease, and 194 (4·9%) of 3958 patients had familial retinoblastoma. The most common cTNMH categories were cT3 (1824 [47·3%] of 3858), N0 (3164 [79·3%] of 3988), M0 (3683 [92·5%] of 3981), and HX (2359 [59·4%] of 3973). These data were available for 94·9% or more patients in the cohort and for 89·2% or more patients in the subanalysis by national income level. Extraocular tumour spread (cT4 of the cTNMH classification) at diagnosis was reported in five (0·8%) of 636 children from high-income countries, 55 (5·4%) of 1027 children from upper-middle-income countries, 342 (19·7%) of 1738 children from lower-middle-income countries, and 196 (42·9%) of 457 children from low-income countries.

The clinical characteristics at presentation, analysed by national income level and data availability, are shown in the appendix (p 3).

Enucleation surgery was available for all patients, and intravenous chemotherapy was available for 4014 (98·8%), being unavailable in six treatment centres in six countries (two low-income countries, two lower-middle-income countries, and two upper-middle-income countries; appendix p 5). Detailed treatment data were available for 4043 (99·5%) patients (appendix pp 4–5). 1937 (47·9%) of 4043 patients received intravenous chemotherapy as primary treatment. Primary enucleation or, rarely, exenteration was done in 1625 (40·2%) of 4043 patients. Primary intraophthalmic artery chemotherapy was done in 304 (7·5%) of 4043 patients, none of whom were from low-income countries. Primary palliative therapy was given in 48 (1·2%) of 4043 children, all from low-income countries and lower-middle-income countries, and upfront treatment refusal was reported in 255 (6·3%) of 4043 patients.

For new or recurring tumours, additional main treatments included intravenous chemotherapy (1311 [32·4%] of 4043), enucleation (1052 [26·0%]), laser or cryotherapy (995 [24·6%]), intra-ophthalmic artery chemotherapy (421 [10·4%]), or intravitreal chemotherapy (343 [8·5%]). Various types of radiotherapy were given to 195 (4·8%) of 4043 patients. Transformation to palliative therapy after initial intent to cure was reported in 18 (0·4%) of 4043 children, and treatment abandonment after initial treatment was reported in 155 (3·8%) patients.

The median follow-up time was 33·2 months (IQR 12·6–39·5), based on 3673 (90·4%) of 4064 reports (table 1). During follow-up, 77 (2·7%) of 2809 patients who presented with unilateral retinoblastoma developed bilateral disease.

Death was reported in 519 (12·8%) of 4064 patients. 146 (30·4%) of 480 patients were from low-income countries, 276 (15·4%) of 1791 were from lower-middle-income countries, 92 (8·0%) of 1151 were from upper-middle-income countries, and five (0·8%) of 642 were from high-income countries (table 1). 472 (90·9%) of 519 deaths were from retinoblastoma and 18 (3·5%) of 519 deaths were from related treatment complications, compared with four (0·8%) of 519 deaths from other causes; for 25 (4·8%) of 519 deaths the cause was not indicated. 307 (59·2%) of 519 deaths followed a diagnosis of metastatic spread.

**Figure 2:**
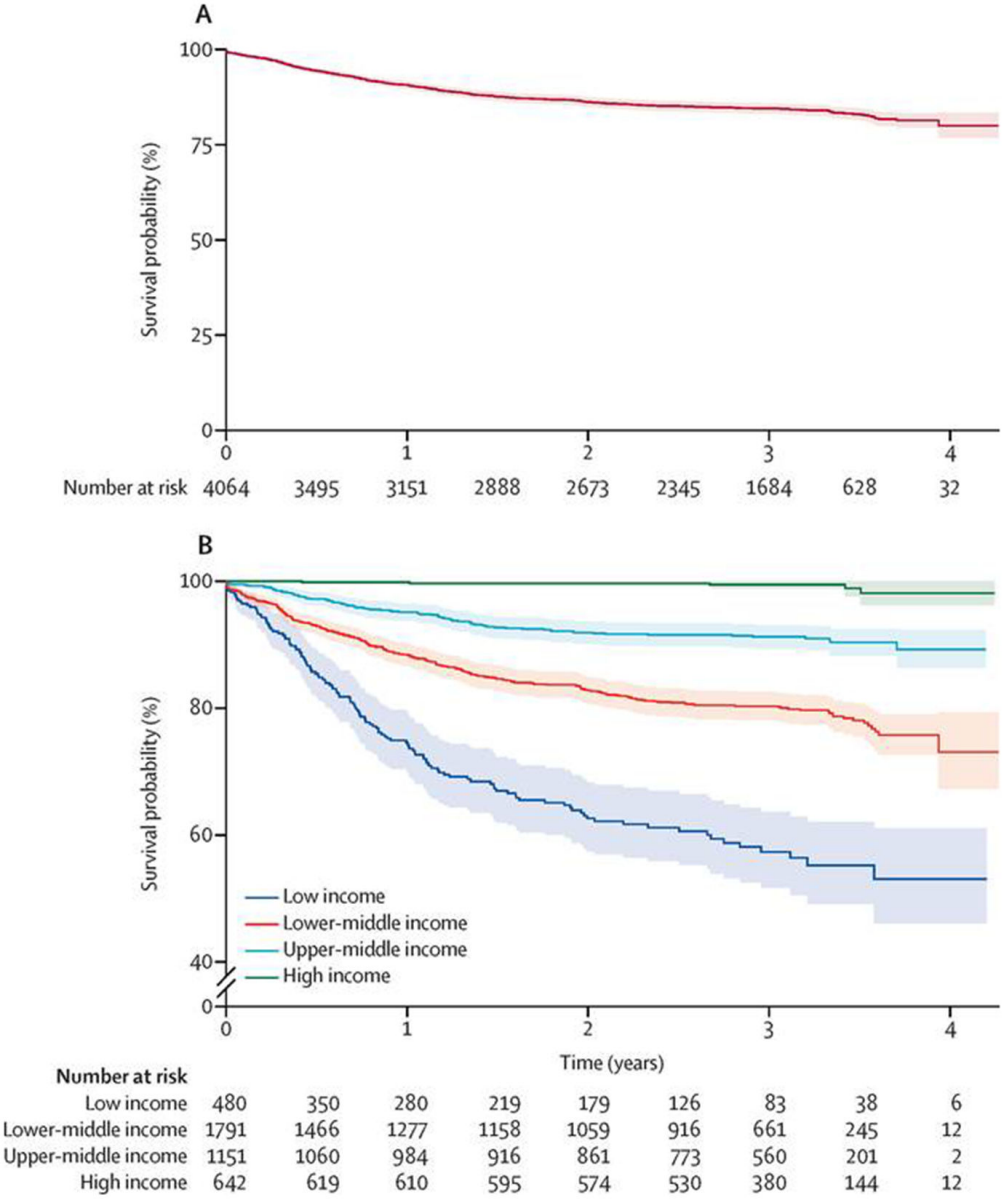
Survival analysis for the full cohort and by income group (A) Kaplan-Meier survival plot for the entire cohort. (B) Kaplan-Meier survival plot by income group.

The Kaplan-Meier survival estimate for the cohort, stratified by national income level, is shown in figure 2. For the entire cohort, the 1-year survival rate was 90·7% (95% CI 89·8–91·6), the 2-year survival rate was 86·2% (85·1–87·3), and the 3-year survival rate was 84·5% (83·3–85·7). The 1-year, 2-year, and 3-year survival rates by national income level and by clinical stage at presentation are shown in the appendix (p 6). In low-income countries, the survival rate declined from 74·4% (95% CI 70·3–78·8) at 1 year to 57·3% (52·1–63·0) at 3 years, in lower-middle-income countries it declined from 88·4% (86·8–90·0) at 1 year to 80·3% (78·3–82·3) at 3 years, in upper-middle-income countries it declined from 95·1% (93·8–96·4) at 1 year to 91·2% (89·5–93·0) at 3 years, whereas in high-income countries it declined from 99·8% (99·5–100·0) at 1 year to 99·5% (98·8–100·0) at 3 years. Overall, for cT1–cT3, the survival rate was 90·5% or higher at 3 years, whereas for cT4 it declined from 55·0% (95% CI 50·8–59·6) at 1 year to 31·9% (27·6–36·9) at 3 years.

Table 2 summarises the results of the clustered and weighted Cox proportional hazards models for survival. Both the income level of the country of residence (low income *vs* high income; hazard ratio 16·67 [95% CI 4·76–50·00]) and extraocular retinoblastoma at diagnosis (cT4 *vs* cT1; 8·98 [4·44–18·18]) were found to be associated with all-cause mortality. On analysis of age at diagnosis, for children aged 0–3 years, the risk of all-cause mortality steadily increased; for children aged 3–7 years, the risk remained almost the same, with a slight decrease over this period (p=0·0104). After 7 years, the all-cause mortality risk steadily decreased; however, the change in the slope was not significant. Sex, familial retinoblastoma, cT2 and cT3 (compared to cT1), laterality, residence in lower-middle-income and upper-middle-income countries (compared to low-income countries), and hereditary retinoblastoma were not significantly associated with survival. Sensitivity analyses showed little change from the primary analysis and no difference in the main conclusions (appendix pp 7–12).

Distant metastasis by 3 years of follow-up was reported in 519 (12·8%) of 4064 patients (not to be confused with the 519 reported deaths), of whom 86 (16·6%) were alive at 3 years. The median time from diagnosis of the primary tumour to diagnosis of metastasis was 3·8 months (IQR 0·2–11·1), based on 260 (50·1%) of 519 patients.

Of the study cohort, 2642 (65·3%) of 4043 underwent enucleation (table 1). Both eyes were enucleated from 122 (3·0%) patients. For the entire cohort, the cumulative incidence of enucleation was 63·1% (95% CI 61·4–64·8) at 1 year, 66·8% (65·1–68·5) at 2 years, and 69·0% (67·4–70·7) at 3 years (appendix p 13).

Table 3 summarises the clustered and weighted Cox proportional hazards model for enucleation. Of the variables included in the analysis, primary tumour category (cT1 compared to cT2, cT3, or cT4) was found to be associated with a lower risk of enucleation as an outcome. On analysis of age at diagnosis, for children aged 0–4 years, the risk of enucleation increased as a function of age, and then steadily decreased (p=0·0039). Children with unilateral retinoblastoma were more likely to have enucleation than children with bilateral disease. Other variables, including national income level, sex, family history of retinoblastoma, and hereditary status did not achieve significance. Sensitivity analyses were done (appendix pp 14–17) as outlined in the Methods, all of which showed little change from the primary analysis.

None of the deaths that occurred during 2020 (20 [3·9%] of 519) and none of the enucleations done during this period (40 [1·5%] of 2677) were associated with the COVID-19 pandemic or a delay in treatment because of the pandemic.

## Discussion

This study shows that a large disparity exists in the survival rate of children with retinoblastoma depending on the economic level of their country of residence. The largest gap, a difference of nearly 17 times, was found between children from high-income countries and low-income countries. In high-income countries, retinoblastoma is considered a curable disease, and death is a rare event, whereas we found that in low-income countries just over 50% of children with retinoblastoma remained alive 3 years after diagnosis.

At the time of diagnosis, 40% of children from low-income countries compared with less than 1% from high-income countries had extraocular tumour spread, and children with an extraocular tumour had a nearly ten-times increased risk of dying compared to those with early intraocular disease. However, low-income status remained a major risk factor for death independently of the stage at diagnosis. Variations in management options by country income level are a possible contributing factor. Enucleation and intravenous chemotherapy, which can save a child’s life, were readily available in nearly all centres and countries. However, we previously reported differences in the availability of sophisticated treatments and investigation facilities in low-income and middle-income countries (such as MRI, targeted chemotherapy, radiotherapy, and focal treatments).^3^ Another possibility is that children in low-income countries present with a more biologically aggressive form of disease, a suggestion that had emerged from our presentation data.^3^

Age at diagnosis independently predicted survival, although its impact was not as significant as the other factors. The risk of death increased until 3 years of age, remained the same for children aged 3–7 years, and then decreased. The latter change in slope, however, was not significant, possibly due to the small number of patients in this age group. Retinoblastoma is believed to develop through a benign retinoma stage, which is typically transient.^16^ It is possible that in children who were diagnosed after the age of 3 years, there was initially a longer retinoma phase, which failed to become fully inactive, gradually becoming larger. At a later age, it eventually escapes the senescence route and begins to develop increasing grades of anaplasia. These tumours might be less advanced than a rapidly transforming retinoma. This hypothesis is speculative, however, and requires further investigation.

The AJCC Ophthalmic Oncology Task Force reported outcomes of patients diagnosed with retinoblastoma in 14 countries.^17^ The 5-year survival rate was 99% for patients residing in high-income countries, 89% for those residing in upper-middle-income countries, and 90% for those residing in lower-middle-income countries. Of the study cohort, 41% of patients were from high-income countries, substantially more than the estimated global incidence in this economic group, and no patients from low-income countries were included. In 2010, a systematic review reported survival after retinoblastoma in 48 low-income and middle-income countries.^2^ Patients from high-income countries were not included, and many of the analysed countries have since migrated their income level classification. Moreover, a survival analysis was not done but instead a global estimate was given for each country without indicating the length of survival. Reported survival rates were 40% in low-income countries, 77% in lower-middle-income countries, and 79% in upper-middle-income countries. We recently did a systematic literature search of the PubMed and EMBASE databases (unpublished data), including clinical case series of retinoblastoma in African countries from Jan 1, 1989, to Dec 31, 2019, and identified only 44 original reports from 24 of 54 countries in Africa during this period. Survival rates ranged widely, from 23% in Zimbabwe to 100% in Egypt. We are not aware of any other large-scale multinational studies that have investigated survival after retinoblastoma.

In the present study, enucleation or exenteration of at least one eye was reported in approximately two-thirds of the cohort, and 3% of patients lost both eyes in an attempt to save their life. In a recent study by the AJCC Ophthalmic Oncology Task Force,^18^ based on a cohort reported earlier,^17^ 55% of patients underwent primary or secondary enucleation. Neither exenteration surgery nor the rate of bilateral enucleation were reported.

In our study, loss of an eye was not related to the income level of the patient’s country. Preserving an eye, even with relatively poor vision, is desirable, but not at the cost of risking the patient’s life. Despite the introduction of new eye-preserving treatment modalities,^19,20^ enucleation remains an important treatment option across the world.

Notably, 2·7% of patients who presented with unilateral disease developed retinoblastoma in the associated eye during follow-up. Careful follow-up of children with unilateral retinoblastoma, including screening for germline *RB1* mutation, is of utmost importance. A family history of retinoblastoma and young age at diagnosis indicates hereditable retinoblastoma and a high chance of bilateralisation in the course of disease. Germline *RB1* mutation status is generally not known at the time of diagnosis when treatment decisions are made. Moreover, genetic testing is not readily available in many countries, and rarely available in low-income countries.^3^

The main factor associated with eye globe salvage was tumour stage at presentation. Children with cT2–T4 retinoblastoma were less likely to have their eye salvaged than children with cT1. The hazard ratios for stages cT2, cT3, and cT4 did not show a gradual increase; rather, the highest hazard ratio was for cT3 (7·51; 95% CI 4·58–12·31). Possible explanations for the relatively lower hazard ratio for children with cT4 tumours are death before enucleation and palliation by chemotherapy only.

Age was another important predictor of eye globe salvage, showing a non-linear relationship with the risk of enucleation, increasing until age 4 years, and then decreasing. Further investigation of the number of cT3 and cT4 cases (data not shown), for which enucleation was a common treatment option, showed no apparent relationship with the age-stratified globe salvage model, suggesting that other factors might be of importance. Further investigation of the relationship between age at diagnosis and risk of enucleation is warranted.

Patients in this study were enrolled in 2017, and treated and followed up until a closing date of March 31, 2021. COVID-19, which was first detected in China in December, 2019,^21^ and subsequently developed into a pandemic, unexpectedly changed medical priorities and policies profoundly throughout the world, potentially affecting patients in our cohort. A previous survey, done in March to April, 2020, involving retinoblastoma specialists from 194 treatment centres in 94 countries, concluded that a shift in health-care resources and COVID-19 pandemic policies across the world might negatively affect children, especially those with new retinoblastomas.^22^ These concerns, however, were not confirmed in the present study, and suggest that our findings might be generalised, and do not represent a biased global outcome of patients with retinoblastoma in response to the COVID-19 pandemic.

The data presented here highlight the importance of the four pillars of the WHO Global Initiative for Childhood Cancer (CURE-ALL): centres of excellence and care networks, universal health coverage, regimens for management, and evaluation.^23^ Improved care networks and universal health coverage will promote early presentation and diagnosis. Expansion of centres of excellence and improved management regimens will address best management practice. Together with monitoring and evaluation, this will improve survival outcomes for children with retinoblastoma in low-income and middle-income settings. Datasets such as this can help establish global norms as called for in the WHO Global initiative for Childhood Cancer and lead to refinement and standardisation of locally appropriate management regimens to improve outcomes. The high response rate and degree of collaboration shown by this project raises the possibility of a live clinical data repository. Such a live collaboration could, in time, provide guidance on evidence-based management.

Our study has several strengths. First, it is, to the best of our knowledge, the largest and most geographically comprehensive study on retinoblastoma outcomes to date. Second, patients were prospectively followed up from presentation for a median time of nearly 3 years, a duration in which most events (ie, death and enucleation) occur.^17^ Third, the data were analysed by methods that take into account clustering, and tested with several sensitivity analyses, showing that the initial methodology selections were appropriate, and that our findings represent real-world outcomes of patients with retinoblastoma.

The main limitation of this study is that this cohort is based on the original convenience sample of the Global Retinoblastoma Presentation Study; however, the original sample comprised an estimated half of new cases that presented to treatment centres in most countries in 2017. We have shown that most patients of the missing half are from low-income and middle-income countries,^3^ and that the capture rate falls within the national income level category. Some of these patients, especially those from low-income countries, never reach a treatment centre,^24^ and they probably stand no chance of survival.^25^ Of the cohort, 45% of patients were girls. On a recent analysis of the global dataset,^26^ no evidence of sex predilection was found, but there was a possibility of gender discrimination in favour of boys who are brought to treatment centres in some parts of Asia. Because of the large size and geographical spread of the cohort, the obtained data related to treatments (available per country and given de facto) were limited to the type of treatment, without further details about the specific treatment protocol, complications, and related side-effects. Similarly, treatment refusal was reported to occur or not, before or after initial treatment was given, with no further details, and therefore no related subanalyses were done. Information about the impact of COVID-19 was limited as it was based on a survey addressing the caregivers in the treatment centres. Children lost to follow-up before the pandemic could potentially have been affected by the pandemic, data for which were not available to us. Nevertheless, it is our assumption that the impact of the COVID-19 pandemic on this cohort of patients was negligible. Age at diagnosis was not linearly linked to survival risk. The best fitting models were used for analysis, but they too were statistical approximations. Age, however, had only a minimal impact compared to the other variables.

In conclusion, according to this prospective global analysis, despite the availability of potentially curative treatment modalities in nearly all countries, profound inequity in mortality from retinoblastoma persists. In high-income countries, nearly 100% of children with retinoblastoma survive, whereas in low-income countries just over 50% remain alive 3 years after diagnosis, if brought at all to receive treatment. Around three in 100 children with retinoblastoma still lose both eyes. Better awareness of the early signs of retinoblastoma, improving access to timely diagnosis, and implementing existing guidelines and treatment recommendations aimed at children in low-income and middle-income countries^27-29^ are crucial to improving retinoblastoma outcomes worldwide. Notably, life-saving treatment was available in nearly all participating centres irrespective of the country of residence. Findings of the present study provide an evidence base for prioritisation and planning for the WHO Global Initiative for Childhood Cancer, which aims to assist governments to support building sustainable, high-quality childhood cancer programmes to improve survival and reduce suffering.

## Supplementary Material

supplemental

## Figures and Tables

**Table 1:** 3-year outcomes in 4064 new patients with retinoblastoma diagnosed in 149 countries in 2017, by national income level

	Low income	Lower-middle income	Upper-middle income	High income	Total
**Enucleation** *					
Yes	349/474 (73·6%); 349/2642 (13·2%)	1197/1783 (67·1%); 1197/2642 (45·3%)	715/1148 (62·3%); 715/2642 (27·1%)	381/638 (59·7%); 381/2642 (14·4%)	2642/4043 (65·3%)
No	125/474 (26·4%); 125/1401 (8·9%)	586/1783 (32·9%); 586/1401 (41·8%)	433/1148 (37·7%); 433/1401 (30·9%)	257/638 (40·3%); 257/1401 (18·3%)	1401/4043 (34·7%)
Total	474/480 (98·8%)	1783/1791 (99·6%)	1148/1151 (99·7%)	638/642 (99·4%)	4043/4064 (99·5%)
**Metastasis** †					
Yes	129/480 (26·9%); 129/519 (24·9%)	267/1791 (14·9%); 267/519 (51·4%)	112/1151 (9·7%); 112/519 (21·6%)	11/642 (1·7%); 11/519 (2·1%)	519/4064 (12·8%)
No	144/480 (30·0%); 144/2729 (5·3%)	1129/1791 (63·0%); 1129/2729 (41·4%)	890/1151 (77·3%); 890/2729 (32·6%)	566/642 (88·2%); 566/2729 (20·7%)	2729/4064 (67·2%)
Unknown	207/480 (43·1%); 207/816 (25·4%)	395/1791 (22·1%); 395/816 (48·4%)	149/1151 (12·9%); 149/816 (18·3%)	65/642 (10·1%); 65/816 (8·0%)	816/4064 (20·1%)
**Survival status** † ‡					
Dead	146/480 (30·4%); 146/519 (28·1%)	276/1791 (15·4%); 276/519 (53·2%)	92/1151 (8·0%); 92/519 (17·7%)	5/642 (0·8%); 5/519 (1·0%)	519/4064 (12·8%)
Alive	334/480 (69·6%); 334/3545 (9·4%)	1515/1791 (84·6%); 1515/3545 (42·7%)	1059/1151 (92·0%); 1059/3545 (29·9%)	637/642 (99·2%); 637/3545 (18·0%)	3545/4064 (87·2%)
**Cause of death**					
Retinoblastoma	140/146 (95·9%); 140/472 (29·7%)	247/276 (89·5%); 247/472 (52·3%)	80/92 (87·0%); 80/472 (16·9%)	5/5 (100·0%); 5/472 (1·1%)	472/519 (90·9%)
Retinoblastoma treatment complication	1/146 (0·7%);1/18 (5·6%)	14/276 (5·1%);14/18 (77·8%)	3/92 (3·3%);3/18 (16·7%)	0	18/519 (3·5%)
Other causes§	2/146 (1·4%);2/4 (50·0%)	1/276 (0·4%);1/4 (25·0%)	1/92 (1·1%);1/4 (25·0%)	0	4/519 (0·8%)
Data missing	3/146 (2·1%); 3/25 (12·0%)	14/276 (5·1%);14/25 (56·0%)	8/92 (8·7%);8/25 (32·0%)	0	25/519 (4·8%)
**Follow-up**					
Follow-up time, months	14·7 (4·9–30·8)	29·5 (7·3–38·7)	35·8 (23·0–40·3)	37·1 (32·6–41·3)	33·2 (12·6–39·5)
Data available	414/480 (86·3%)	1598/1791 (89·2%)	1038/1151 (90·2%)	623/642 (97·0%)	3673/4064 (90·4%)

Data are n/N (%) or median (IQR). Percentages within the national income level and within the evaluated variable are shown.

* Per patient, enucleation or exenteration, primary or secondary, one or both eyes.

† Data were completed for all study patients.

‡ Trilateral retinoblastoma was reported in seven (1·3%) of 519 patients.

§ Other causes of death were trauma, cardiac arrest, intestinal obstruction, and malaria.

**Table 2:** Cluster-weighted Cox proportional hazards models for survival at 3 years in 4064 new patients with retinoblastoma diagnosed in 149 countries in 2017*

	Coefficient	Robust standard error	Z_score_	p value (corrected†)	HR (95% CI)
**Income level of country of residence**					
Low income	Ref	..	..	..	1·00
Lower-top income	−0·301	0·207	−1·455	0·146 (0·99)	0·73 (0·49–1·11)
Upper-top income	−0·622	0·260	−2·397	0·017 (0·221)	0·54 (0·32–0·89)
High income	−2·821	0·651	−4·330	<0·0001 (0·0002)	0·06 (0·02–0·21)
**All ages** ‡					
HR per month	0·027	0·005	4·941	<0·0001 (<0·0001)	1·03 (1·02–1·04)
HR per year	0·324	0·06	4·941	<0·0001 (<0·0001)	1·38 (1·23–1·56)
**Age >3 years**					
HR per month	−0·028	0·008	−3·368	0·0008 (0·0104)	0·97 (0·96–0·99)
HR per year	−0·336	0·096	−3·368	0·0008 (0·0104)	0·71 (0·59–0·86)
**Age >7 years**					
HR per month	−0·021	0·016	−1·315	0·188 (0·99)	0·98 (0·95–1·01)
HR per year	−0·252	0·192	−1·315	0·188 (0·99)	0·78 (0·53–1·13)
**Bilaterality**					
Unilateral	Ref	..	..	..	1·00
Bilateral	0·471	0·567	0·831	0·41 (0·99)	1·60 (0·53–4·87)
**Primary tumour**					
cT1	Ref	..	..	..	1·00
cT2	0·065	0·295	0·221	0·825 (0·99)	1·07 (0·60–1·90)
cT3	0·171	0·336	0·509	0·611 (0·99)	1·19 (0·61–2·29)
cT4	2·196	0·360	6·104	<0·0001 (<0·0001)	8·98 (4·44–18·18)
**Sex**					
Male	Ref	··	··	··	1·00
Female	0·129	0·077	1·669	0·095 (0·99)	1·14 (0·98–1·32)
**Family history of retinoblastoma**					
Yes	Ref	..	..	..	1·00
No	0·182	0·333	0·546	0·585 (0·99)	1·20 (0·62–2·31)
**Hereditary retinoblastoma**					
Non-hereditary	Ref	..	..	..	1·00
Hereditary§	−0·265	0·585	−0·454	0·650 (0·99)	0·77 (0·24–2·41)

HR=hazard ratio.

* Overall, 500 observations were dropped from the survival analysis because of missing observation time.

† Multiplied by 13, according to Bonferroni’s model.

‡ Age included in the analysis as a continuous variable. Further details of the relationship between age and log hazard for both survival and eye globe salvage are provided in the appendix (pp 1–2).

§ Hereditary refers to bilateral or trilateral retinoblastoma, positive family history, or positive blood *RB1* mutation (H1 in cTNMH).

**Table 3:** Cluster-weighted Cox proportional hazards models for eye globe salvage at 3 years in 4064 new patients with retinoblastoma diagnosed in 149 countries in 2017*

	Coefficient	Robust standard error	Z_score_	p value (corrected†)	HR (95% CI)
**Income level of country of residence**					
Low income	Ref	..	..	..	1·00
Lower-middle income	−0·178	0·116	−1·527	0·127 (0·99)	0·84 (0·67–1·05)
Upper-middle income	−0·353	0·202	−1·747	0·08 (0·99)	0·70 (0·47–1·04)
High income	−0·166	0·171	−0·971	0·332 (0·99)	0·85 (0·61–1·18)
**All ages** ‡					
HR per month	0·007	0·002	3·322	0·0009 (0·0117)	1·01 (1·00–1·01)
HR per year	0·084	0·024	3·322	0·0009 (0·0117)	1·09 (1·04–1·14)
**Age >4 years**					
HR per month	−0·012	0·003	−3·598	0·003 (0·034)	0·99 (0·98–1·00)
HR per year	−0·144	0·036	−3·598	0·003 (0·034)	0·87 (0·81–0·93)
**Laterality**					
Unilateral	Ref	..	..	..	1·00
Bilateral	−0·428	0·142	−3·010	0·002 (0·026)	0·65 (0·49–0·86)
**Primary tumour**					
cT1	Ref	..	..	..	1·00
cT2	1·024	0·226	4·529	<0·0001 (<0·0001)	2·78 (1·79–4·34)
cT3	2·021	0·252	8·027	<0·0001 (<0·0001)	7·54 (4·61–12·36)
cT4	1·510	0·318	4·748	<0·0001 (<0·0001)	4·53 (2·43–8·45)
**Sex**					
Male	Ref	..	..	..	1·00
Female	0·077	0·059	1·290	0·197 (0·99)	1·08 (0·96–1·21)
**Family history of retinoblastoma**					
Yes	Ref	..	..	..	1·00
No	0·299	0·178	1·678	0·093 (0·99)	1·35 (0·95–1·91)
**Hereditary retinoblastoma**					
Non-hereditary	Ref	..	..	..	1·00
Hereditary§	−0·121	0·124	−0·975	0·330 (0·99)	0·89 (0·70–1·13)

HR=hazard ratio.

* Overall, 797 observations were dropped from the eye globe salvage analysis because of missing observation time.

† Multiplied by 13, according to Bonferroni’s model.

‡ Age included in the analysis as a continuous variable. Further details of the relationship between age and log hazard for both survival and eye globe salvage are provided in the appendix (pp 1–2).

§ Hereditary refers to bilateral or trilateral retinoblastoma, positive family history, or positive blood *RB1* mutation (H1 in cTNMH).

## Data Availability

The study data will become available online once all analyses are complete.

## Research in context

### Evidence before this study

A literature search was done on PubMed from database inception to October, 2020, with the search terms “retinoblastoma”; and country name (eg, “Germany”) according to the World Bank list of countries. No date or language restrictions were applied. We included all original studies in which patients with retinoblastoma were evaluated. Publications were identified from 92 countries, with the vast majority of studies being from single retinoblastoma centres. Three articles by the American Joint Committee on Cancer Ophthalmic Oncology Task Force, all using the same cohort of patients, were published in 2020, comparing the outcome of treatment for retinoblastoma from 14 countries with different national income levels, none of which were low-income countries. Compared with high-income countries, mortality was 10·3-times higher for upper-middle-income countries and 9·3-times higher for lower-middle-income countries. We found no study investigating retinoblastoma outcomes on a global scale. Using PubMed and EMBASE, we also did a systematic review of all original studies reporting data on patients with retinoblastoma in African countries from Jan 1, 1989, to Dec 31, 2019. We used no language barriers. Following removal of duplicate entries, and screening of titles, abstracts, and the full text, we identified 44 original manuscripts from 24 of 54 countries in Africa. Reported survival rates ranged from 23% (Zimbabwe) to 100% (Egypt).

### Added value of this study

This prospective cluster-based analysis reports 3-year outcomes of 4064 treatment-naive patients with retinoblastoma recruited in 2017 from 149 countries, including 41 African countries. It is estimated that this cohort represents approximately half of all new children diagnosed with retinoblastoma worldwide in 2017. Our findings show that in high-income countries retinoblastoma is a curable disease with nearly 100% survival, whereas in low-income countries survival after 3 years is just over 50%. Life-saving treatments were reported to be available in nearly all countries. Risk factors for this huge disparity in survival included the income level of the country of residence (low income *vs* high income), extraocular retinoblastoma at diagnosis, and older age at diagnosis (up to age 3 years).

### Implications of all the available evidence

This study reports a huge global inequity in the prognosis of retinoblastoma in children. It provides evidence for action at national and international levels, including the WHO Global Initiative for Childhood Cancer, which aims to provide leadership and technical assistance to governments to develop high-quality childhood cancer programmes to improve survival and reduce suffering.

## The Global Retinoblastoma Study Group

*Albania:* Donjeta B Alia, Alketa Tandili (University Hospital Centre “Mother Theresa”, Tirana). *Angola:* Luisa Paiva, Amelia DC Wime (National Ophthalmological Institute of Angola, Luanda). *Argentina:* Guillermo L Chantada, Adriana C Fandiño, Mariana Sgroi (Hospital JP Garrahan, Buenos Aires), Guillermo L Chantada (Scientific and Technical Research Council, CONICET, Buenos Aires). *Armenia:* Ruzanna Papyan, Gevorg Tamamyan (Yerevan State Medical University, Department of Oncology and Pediatric Cancer and Blood Disorders Center of Armenia, Hematology Center after R.H. Yeolyan, Yerevan). *Australia:* Jayne E Camuglia, Glen A Gole (Department of Ophthalmology, Queensland Children’s Hospital, Brisbane, Queensland), Antony Clark, Geoffrey C Lam (University of Western Australia, Perth Children’s Hospital, Perth, Western Australia), James E Elder, John D McKenzie, Sandra E Staffieri (Department of Ophthalmology, Royal Children’s Hospital, Parkville, Victoria), James E Elder (Department of Paediatrics, University of Melbourne, Parkville, Victoria), Michael M Jones (The Children’s Hospital at Westmead, Sydney, New South Wales), John D McKenzie (Department of Ocular Oncology, Royal Victorian Eye and Ear Hospital, East Melbourne, Victoria), Sandra E Staffieri (Center for Eye Research Australia, University of Melbourne, East Melbourne, Victoria), Rebecca Manudhane, David Sia (Women’s and Children’s Hospital, North Adelaide, South Australia), David Sia (Discipline of Ophthalmology and Visual Sciences, University of Adelaide and South Australian Institute of Ophthalmology, Adelaide, South Australia). *Austria:* Petra Ritter-Sovinz (Department of Pediatrics and Adolescent Medicine, Division of Pediatric Hematology/Oncology, Medical University of Graz, Graz), Christoph Schwab (Department of Ophthalmology, Medical University Graz, Graz). *Azerbaijan:* Ruhengiz Balayeva (Zarifa Aliyeva National Centre of Ophthalmology, Baku). *Bangladesh:* Zohora Khan (Dhaka Medical College Hospital, Dhaka), Murtuza Nuruddin, Soma R Roy (Chittagong Eye Infirmary & Training Complex, Chittagong), Riffat Rashid, Sadia Sultana (Department of Oculoplasty and Ocular Oncology, Ispahani Islamia Eye Institute and Hospital, Dhaka), Shawkat A Shakoor (National Institute of Ophthalmology, Dhaka). *Belarus:* Larisa Naumenko, Katsiaryna Zhilyaeva (N.N. Alexandrov National Cancer Centre of Belarus, Minsk). *Belgium:* Paulina Bartoszek, Bénédicte G Brichard, Patrick De Potter (Cliniques Universitaires Saint-Luc, Brussels). *Benin:* Amadou I Alfa Bio (University of Parakou, Parakou). *Bolivia:* Beatriz Salas (Hospital Dr. Manuel Ascencio Villarroel, Cochabamba), Maria Estela Coleoni Suarez (Pediatra Hemato-Oncologa, Instituto Oncologico del Oriente Boliviano, Santa Cruz de la Sierra). *Botswana:* Freddy B Mbumba (Botswana Government - Scottish Livingstone Hospital, Molepolole). *Brazil:* Maria Teresa BC Bonanomi (Hospital das Clínicas da FMUSP, São Paulo), Carla R Donato Macedo (Pediatric Oncology Institute/GRAACC, Federal University of São Paulo, São Paulo), Nathalia DAK Grigorovski, Clarissa CDS Mattosinho (National Institute of Cancer in Brazil, Rio de Janeiro), Luiz F Teixeira (Ophthalmology Department, Federal University of São Paulo, São Paulo), Luiz F Teixeira (Pediatric Oncology Institute, Federal University of São Paulo, São Paulo). *Bulgaria:* Alexander H Oscar, Nevyana V Veleva-Krasteva (Eye Clinic, University Hospital “Alexandrovska”, Department of Ophthalmology, Medical University, Sofia). *Burkina Faso:* Gabrielle C Bouda, Rolande L Kabore (Centre Hospitalier Universitaire Yalgado Ouédraogo de Ouagadougou, Ouagadougou). *Burundi:* Remezo Philbert (Centre Hospitaliere Universitaire de Kamenge, Bujumbura). *Cameroon:* Ted Grimbert A Evina, Henry E Nkumbe (Magrabi ICO Cameroon Eye Institute, Yaounde), Pius Kamsang, Okwen M Muyen (Abii Specialists Hospital, Bamenda). *Canada:* Helen Dimaras, Ashwin Mallipatna (The Hospital for Sick Children, Toronto), Patrick Hamel, Rosanne Superstein (CHU Sainte Justine, University of Montreal, Montréal), Katherine E Paton (University of British Columbia, Vancouver, British Columbia), Caron Strahlendorf (BC Children’s Hospital, Vancouver). *Central African Republic:* Jess Elio Kosh Komba Palet (Oncologue Pédiatre Responsable d’Unité de Bangui, Bangui). *Chad:* Harba Tyau-Tyau (Université Adam Barka, Adam Barka). *Chile:* Isabel Cavieres, Juan P López (Ophthalmology Department, Universidad de Chile, Santiago), Joaquin Oporto, Diego Ossandon (Clínica Alemana de Santiago, Universidad del Desarrollo, Santiago). *China:* Wensi Chen, Daoman Xiang (Department of Pediatric Ophthalmology, Guangzhou Children’s Hospital and Guangzhou Women and Children’s Medical Center, Guangzhou Medical University, Guangzhou), Yi Du, Kaijun Li (Department of Ophthalmology, the First Affiliated Hospital of Guangxi Medical University, Nanning), Xunda Ji, Jing Tang (Department of Ophthalmology, Xinhua Hospital, Shanghai Jiao Tong University School of Medicine, Shanghai), Cairui Li, Bing Xu (The first hospital of Dali University, Yunnan Province), Jiang Qian, Kang Xue (Department of Ophthalmology, Eye and ENT Hospital of Fudan University, Shanghai), Xiantao Sun (Henan Children’s Hospital, Affiliated Children’s Hospital of Zhengzhou University, Zhengzhou), Yi-Zhuo Wang, Yi Zhang (Department of Paediatrics, Beijing Tongren Hospital, Capital Medical University, Beijing), Si-qi Wu, Yishuang Xiao (Kunming Children’s Hospital, Kunming), Huasheng Yang, Huijing Ye (State Key Laboratory of ophthalmology, Zhongshan Ophthalmic Center, Sun Yat-sen University, Guangzhou). *Colombia:* Rodrigo A Polania (Fundacion Clinica Valle del Lili, Cali). *Côte d’Ivoire:* Rokia C Berete (Ophthalmologic Department of the Teaching Hospital of Treichville, Abidjan), Line Couitchere (Pediatric Unit, Teaching Hospital of Treichville, Félix Houphouët Boigny University, Abidjan). *Croatia:* Sanja Perić (University Hospital Centre Zagreb, Zagreb). *Cuba:* Ernesto Alemany-Rubio, Liudmira González-Rodríguez (Instituto Cubano de Oftalmología “Ramón Pando Ferrer”, Marianao, Havana). *Czech Republic:* Rudolf Autrata (Pediatric Ophthalmology Department, Children’s Hospital, Faculty of Medicine, Masaryk University Hospital Brno, Brno), Tomas Kepak (University Hospital Brno and St. Anna University Hospital/ICRC, Masaryk University, Brno), Pavel Pochop (Department of Ophthalmology for Children and Adults, 2nd Faculty of Medicine, Charles University in Prague and Motol University Hospital, Prague), Karel Svojgr (Department of Pediatric Hematology and Oncology, 2nd Faculty of Medicine, Charles University in Prague and Motol University Hospital, Prague). *Denmark:* Pernille A Gregersen (Department of Clinical Genetics, and Centre for Rare Disorders, Aarhus University Hospital, Aarhus), Steen F Urbak (Department of ophthalmology, Aarhus University Hospital, Aarhus). *Dominican Republic:* Margarita M Montero (Hospital Infantil Dr. Robert Reid Cabral, Santo Domingo). *Democratic Republic of the Congo:* Aléine Budiongo, Jenny M Yanga (Cliniques Universitaires de Kinshasa, Université de Kinshasa, Kinshasa), Theophile B Kabesha Amani (Bukavu Eye Clinic - Bukavu Official University, Bukavu), Robert M Lukamba, Marcel N Numbi (University Clinics of Lubumbashi, University of Lubumbashi, Lubumbashi). *Ecuador:* Doris A Calle Jara, Edwin G Villacís Chafla (Hospital del Niño Dr. Francisco De Icaza Bustamante, Guayaquil), Gissela L Sánchez (Hospital Solca Quito, Quito). *Egypt:* Sherif Abouelnaga, Mahmoud A Afifi, Alaa M Elhaddad (Children’s Cancer Hospital Egypt 57357, Cairo), Amany M Ali, M Elzembely (Pediatric Oncology Department, South Egypt Cancer Institute, Assiut University, Assiut), Azza MA Said, Othman AO Ziko (Ophthalmology Department, Faculty of Medicine, Ain Shams University, Cairo). *ElSalvador:* Soad L Fuentes-Alabi, Marco A Goenz (Pediatric Oncology Department, National Children’s Hospital Benjamin Bloom, San Salvador). *Estonia:* Katrin Eerme, Artur Klett (East Tallinn Central Hospital, Tallinn). *Ethiopia:* Diriba F Hordofa (Department of Pediatrics and Child Health, Jimma University Medical Center, Jimma), Aemero A Mengesha (Department of Ophthalmology, Jimma University, Jimma), Sadik T Sherief (Addis Ababa University, School of Medicine, Department of Ophthalmology, Addis Ababa). *Finland:* Tero T Kivela, Kalle Nummi (Ocular Oncology Service, Department of Ophthalmology, University of Helsinki and Helsinki University Hospital, Helsinki). *France:* Nathalie Cassoux (Institut curie, université de Paris medicine Paris V Descartes, Paris), Laurence Desjardins (Institut curie, Paris). *Gabon:* Ghislaine Obono-Obiang (Chu Angondje Cancerologie, Libreville). *Georgia:* Tamar Kardava, Zaza Khotenashvili (LTD ‘’New Hospitals”, Tbilisi). *Germany:* Nikolaos E Bechrakis, Eva M Biewald, Sabrina Schlüter (University Hospital Essen, Department of Ophthalmology, University Duisburg-Essen, Essen), Petra Ketteler (Department of Pediatrics III, University Hospital Essen, University Duisburg-Essen, Essen). *Ghana:* Doreen Amankwaa-Frempong (Department of Ophthalmology, Komfo Anokye Teaching Hospital, Kumasi), Vera A Essuman (Ophthalmology Unit, Department of Surgery, University of Ghana Medical School, University of Ghana, Accra), Vivian Paintsil (School of Medicine and Dentistry, Kwame Nkrumah University of Science and Technology, Komfo Anokye Teaching Hospital, Kumasi), Lorna A Renner (Department of Child Health, University of Ghana Medical School, University of Ghana, Accra). *Guatemala:* Amanda Alejos, Ana V Girón (Unidad Nacional de Oncología Pediátrica, Guatemala City). *Haiti:* Yvania Alfonso Carreras (St. Damien Pediatric Hospital, Port-au-Prince). *Honduras:* Ligia D Fu, Carlos Maldonado (Hospital Escuela, *Tegucigalpa).* *Hong Kong Special Administrative Region, China:* Emily S Wong, Jason C Yam (Hong Kong Eye Hospital, Chinese University of Hong Kong, Hong Kong). *Hungary:* Monika Csóka, Erika Maka (Semmelweis University Budapest, Budapest). *India:* Priyanka Aggarwal, Vineeta Gupta (Department of Pediatrics, Banaras Hindu University, Varanasi), Anirban Bhaduri (Susrut Eye Foundation & Research Institute, Kolkata), Arpita Bhattacharyya, Anirban Das (Department of Pediatric Hematology Oncology, Tata Medical Center, Kolkata), Bhavna Chawla (Ocular Oncology Service, Dr. Rajendra Prasad Centre for Ophthalmic Sciences, All India Institute of Medical Sciences, New Delhi), Pranab Das (The Calcutta Medical Research Institute, Kolkata), Sima Das (Ocular Oncology Services, Dr Shroff’s Charity Eye Hospital, New Delhi), Himika Gupta (Bai Jerbai Wadia Hospital for Children, Mumbai), Sanjiv Gupta, Nishant Verma (King George’s Medical University, Lucknow, Uttar Pradesh), Swathi Kaliki (The Operation Eyesight Universal Institute for Eye Cancer, L V Prasad Eye Institute, Hyderabad), Vikas Khetan, Puja Maitra (Sankara Nethralaya, Chennai), Amita Mahajan (Pediatric Hematology-Oncology Unit, Apollo Center for Advanced Pediatrics, Indraprastha Apollo Hospital, New Delhi), Vikas Menon (Centre for Sight, New Delhi), Divyansh KC Mishra, Mahesh Shanmugam Palanivelu, Rajesh Ramanjulu (Sankara Eye Hospital, Bangalore), Sangeeta S Mudaliar (Bai Jerbai Wadia Hospital for Children, Mumbai), Akshay Gopinathan Nair, Sundaram Natarajan (Aditya Jyot Eye Hospital, Mumbai), Akshay Gopinathan Nair (Lokmanya Tilak Municipal Medical College and General Hospital, Mumbai), Rachna Seth (Department of Pediatrics, All India Institute of Medical Sciences, New Delhi), Usha Singh (Department of Ophthalmology, Postgraduate Institute of Medical Education and Research, Chandigarh), Sunil Bhat, Gagan Dudeja (Department of Pediatric Hematology and Oncology, Narayana Health City, Bangalore), Devjyoti Tripathy (LV Prasad Eye Institute, MTC Campus, Bhubaneswar, Odisha), Indonesia Marliyanti NR Akib, Halimah Pagarra (RS Dr. Wahidin Sudirohusodo, Makassar), Primawita O Amiruddin, Mayasari W Kuntorini (National Eye Center-Cicendo Eye Hospital, Bandung), Inggar Armytasari, Eddy Supriyadi (Sardjito Hospital-Faculty of Medicine, Universitas Gadjah Mada, Yogyakarta), I Wayan Eka Sutyawan, Putu Yuliawati (Departement of Opthalmology Udayana University, Sanglah Eye Hospital, Bali), Delfitri Lutfi, Hendrian D Soebagjo (Ophthalmology Department, Airlangga University - Dr. Soetomo General Hospital, Surabaya), Ardizal Rahman (Ophthalmology Department, Dr M Djamil General Hospital, Medical Faculty Andalas University West Sumatra), Rita S Sitorus, Andi A Victor (Department of Ophthalmology, Faculty of Medicine Universitas Indonesia - Dr. Cipto Mangunkusumo National General Hospital, Jakarta), Edi S Tehuteru, Widiarti Widiarti (National Cancer Center - “Dharmais” Cancer Hospital, Jakarta), Yetty M Nency (Child Health Department, Faculty of Medicine, Diponegoro University, Semarang). *Iran:* Mohammad Faranoush (Pediatric Growth and Development Research Center, Institute of Endocrinology, Iran University of Medical Sciences, Rasool Akram Hospital, Tehran), Azim Mehrvar, Maryam Tashvighi (Mahak childrens Hematology Oncology Research Center (Mahak-HORC), Mahak Hospital, Tehran), Ahad Sedaghat (Eye Research Center, The Five Senses Institute, Rassoul Akram Hospital, Iran University of Medical Sciences, Tehran), Fariba Ghassemi, Alireza Khodabande (Retina & Vitreous Service, Farabi Eye Hospital, Tehran University of Medical Sciences, Tehran). *Iraq:* Rula A Abdulqader, Athar ASM Al-Shaheen (Basra Children Specialty Hospital, Basra), Mouroge H Al Ani, Huda Haydar (Hawler Medical University, Erbil), Safaa AF Al-Badri, Mazin F Al-Jadiry, Ahmed H Sabhan (Pediatric Oncology Unit, Children Welfare Teaching Hospital, Medical City, College of Medicine, University of Baghdad, Bagdad), Usama Al-Jumaily (Imam Hussein Cancer Center, Kerbala), Ali ARM Al-Mafrachi (Ibn AlHaitham Teaching Eye Hospital, Baghdad), Entissar H Al-Shammary, Allawi N Hussein Al-Janabi (Oncology Unit, Child Central Teaching Hospital , Baghdad), Ali O Qadir (Hiwa Cancer Hospital, Al Sulaimaniyah). *Ireland:* Michael Capra (Our Lady’s Children’s Hospital, Dublin). *Israel:* Sharon Blum, Nir Gomel (Tel Aviv Sourasky Medical Center and The Sackler Faculty of Medicine, Tel-Aviv University, Tel-Aviv), Ido Didi Fabian, Hila Goldberg, Noa Kapelushnik, Shiran Madgar, Victoria Vishnevskia-Dai (Goldschleger Eye Institute, Sheba Medical Center, Tel Hashomer, Tel-Aviv University, Tel-Aviv), Shahar Frenkel, Jacob Pe’er (Hadassah Hebrew University Medical Center, Jerusalem), Malka Gorfine, David Refaeli, David M Steinberg (Department of Statistics and Operations Research, School of Mathematical Sciences, Raymond and Beverly Sackler Faculty of Exact Sciences, Tel Aviv University, Tel Aviv), Yotam Lavy (Department of Ophthalmology, Soroka University Medical Center, Beer Sheva), Helen Toledano (Department of Pediatric Hematology-Oncology, Schneider Children’s Medical Center, Sackler School of Medicine, Tel-Aviv University), Shani Caspi (Pediatric Oncology, Sheba Medical Center, Tel Aviv University, Tel Aviv). *Italy:* Sonia De Francesco, Theodora Hadjistilianou (Retinoblastoma referral center, University of Siena, Siena), Russo Ida, Paola Valente (Bambino Gesù IRCCS Children’s Hospital, Rome), Edoardo Midena, Raffaele Parrozzani (Department of Ophthalmology, University of Padova, Padova). *Jamaica:* Kristin E Cowan-Lyn, Leon O Vaughan (Bustamante Hospital for Children, Kingston). *Japan:* Shigenobu Suzuki (Department of Ophthalmic Oncology, National Cancer Center Hospital, Tokyo). *Jordan:* Mona T Mohammad, Yacoub A Yousef (King Hussein Cancer Center, Amman). *Kazakhstan:* Lyazat Manzhuova (Scientific Centre for Pediatrics and Pediatric Surgery, Almaty). *Kenya:* Rose Atsiaya, Ibrahim O Matende (Light House for Christ Eye Centre, Mombasa). *Kyrgyzstan:* Ainura S Begimkulova, Emil K Makimbetov (National Center of Oncology and Hematology, Bishkek). *Laos:* Jonny Keomisy, Phayvanh Sayalith (Mahosot Hospital, Vientiane). *Latvia:* Sandra Valeina, Maris Viksnins (Children’s Clinical University Hospital, Riga). *Lebanon:* Christiane E Al-Haddad (Department of Ophthalmology, American University of Beirut Medical Center, Beirut), Raya H Saab (Children’s Cancer Institute, American University of Beirut Medical Center, Beirut), *Libya:* Khalifa M Alsawidi, Amal M Elbahi (Tripoli Eye Hospital, Tripoli University, Tripoli). *Lithuania:* Dalia Krivaitiene (Chidren’s Ophthalmology Department, Chidren’s Hospital of Vilnius, University Hospital Santaros Clinic, Vilnius). *Macedonia:* Bekim Tateshi (University Eye Clinic, Skopje). *Madagascar:* Hoby Lalaina Randrianarisoa, Léa Raobela (Centre Hospitalier Universitaire Joseph Ravoahangy Andrianavalona, Antananarivo). *Malawi:* Gerald Msukwa, Chinsisi Nyirenda (Lions Sight First Eye Hospital, Queen Elizabeth Central Hospital, Blantyre). *Malaysia:* Norhafizah Hamzah, Kok Hoi Teh (Hospital Kuala Lumpur, Kuala Lumpur). *Mali:* Fatoumata Sylla (Africa Institute of Tropical Ophtalmology, Bamako), Fousseyni Traoré (Pediatric Oncology Service, Gabriel Toure Hospital, Bamako). *Mauritania:* Sidi Sidi cheikh (Ophthalmology department, Nouakchott Medical University, Nouakchott), Ekhtelbenina Zein (Assistante Hospitalo - Universitaire, Faculte de Medecine de Nouakchott Medecin Oncopediatre, Centre National d’Oncologie, Nouakchott). *Mexico:* Graciela Gonzalez Perez, Alma Janeth Sanchez Orozco (Hospital Civil de Guadalajara, Guadalajara), Miriam Ortega-Hernández, Marco A Ramirez-Ortiz (Department of Ophthalmology Hospital Infantil de Mexico Federico Gómez, Mexico City). *Mongolia:* Tsengelmaa Chuluunbat (National Center for Maternal and Children Health of Mongolia, Ulaanbaatar). *Morocco:* Elhassan Abdallah (Ophthalmology Department of Rabat, Mohammed V University, Rabat), Sarra Benmiloud (Department of Pediatric Oncology, University Hassan II Fès, Fez), Asmaa El Kettani (Centre Hospitalier et Universitaire Ibn Rochd, faculté de médecine et de pharmacie Université Hassan 2, Casablanca), Laila Hessissen (Pediatric Hematology and Oncology Department of Rabat - Mohammed V University, Rabat). *Mozambique:* Argentino A Almeida (Beira Central Hospital, Beira). *Nepal:* Ben Limbu, Purnima Rajkarnikar, Rohit Saiju (Tilganga Institute of Ophthalmology, Kathmandu). *Netherlands:* Annette C Moll, Milo van Hoefen Wijsard (Department of Ophthalmology, Amsterdam University Medical Centers, Amsterdam). *New Zealand:* Ruellyn L Cockcroft, Yvonne Ng (Starship Children’s Hospital, Auckland), Andrew J Dodgshun (Department of Paediatrics, University of Otago (Christchurch), Children’s Haematology/Oncology Centre, Christchurch Hospital, Christchurch). *Nicaragua:* Patricia Calderón-Sotelo (Hospital Infantil Manuel de Jesús, Managua). *Nigeria:* Shehu U Abdullahi, Sadiq Hassan, Ali B Umar (Bayero University, Aminu Kano Teaching Hospital, Kano), Aminatu A Abdulrahaman, Amina H Wali (National Eye Centre Kaduna, Kaduna), Dupe S Ademola-Popoola (University of Ilorin & University of IlorinTeaching Hospital, Ilorin, Kwara State), Adedayo Adio (Department of Ophthalmology, University of Port Harcourt Teaching Hospital, Port Harcourt), Ada E Aghaji, Ifeoma R Ezegwui (Department of Ophthalmology, College of Medicine, University of Nigeria, Enugu), Adeseye Akinsete, Kareem O Musa (Department of Ophthalmology, Lagos University Teaching Hospital/College of Medicine of the University of Lagos, Lagos), Oluyemi Fasina (Department of Ophthalmology, University College Hospital/University of Ibadan, Ibadan, Oyo State), Affiong Ibanga, Elizabeth D Nkanga (Calabar Children’s Eye Centre, Department of Ophthalmology University of Calabar Teaching Hospital Calabar Cross River State, Calabar), Tajudeen Mustapha, Dahiru Ribadu (Federal Medical Centre, Yola). *Norway:* Marlies Hummelen (Eye Department, Oslo university hospital, Oslo). *Pakistan:* Alia Ahmad, Asma Mushtaq, Seema Qayyum (The Children’ Hospital & the Institute of Child Health, Lahore), Shabana Chaudhry (Paediatric Ophthalmology Department, Mayo Hospital & College of Allied Visual Sciences [COAVS], King Edward Medical University, Lahore), Zehra Fadoo, Irfan Jeeva, Sidra Masud (Aga Khan University, Karachi), Syed A Hamid, Nida Zia (The Indus Hospital, Karachi), Shadab Hassan, Sorath Noorani Siddiqui (Department of Pediatric Ophthalmology and Strabismus, Al Shifa Trust Eye Hospital, Rawalpindi), Teyyeb Janjua, Muhammad A Yaqub (Armed Forces Institute of Ophthalmology, Rawalpindi), Hussain A Khaqan (Department of Ophthalmology, Post Graduate Medical Institute, Ameer Ud Din Medical College, Lahore General Hospital, Lahore). *Panama:* Karina Quintero D, Roberto I Yee (Hospital del Niño “Dr. José Renán Esquivel”, Panama City). *Papua New Guinea:* Vivekaraj Jairaj (Pacific International Hospital, Port Moresby). *Paraguay:* Miriam R Cano (Eye Health National Program, Ministry of Public Health, Asunción, Py), Delia DPG Fernández (MICLINIC, Ciudad del Este). *Peru:* Rosdali Y Díaz Coronado, Arturo M Zapata López (Instituto Nacional de Enfermedades Neoplasicas, Lima), Juan L Garcia, Jimena Ponce (Anglo American Clinic, Lima), Henry N Garcia Pacheco (Pediatric Oncology Unit, Instituto Regional de Enfermedades Neoplásicas del Sur - IREN SUR, Arequipa), Claudia R Pascual Morales, Jacqueline Karina Vasquez Anchaya (Hospital Nacional Guillermo Almenara Irigoyen, Lima), Fanny F Tarrillo Leiva (Hospital Nacional Edgardo Rebagliati Martins, Lima). *Philippines:* Ana Patricia A Alcasabas, Gary John V Mercado (University of the Philippines - Philippine General Hospital, Manila). *Poland:* Krzysztof Cieslik, Wojciech Hautz, Anna Rogowska (Department of Ophthalmology, The Children’s Memorial Health Institute, Warsaw). *Portugal:* Guilherme Castela, Sónia Silva (Centro Hospital Universitário de Coimbra, University of Coimbra, Cioimbra). *South Korea:* Dong Hyun Jo (Department of Anatomy and Cell Biology, Seoul National University College of Medicine, Seoul), Jeong Hun Kim (Department of Ophthalmology, Seoul National University Hospital, Seoul). *Romania:* Codruta Comsa, Monica D Dragomir (Oncology Institute “Prof. Dr. Al. Trestioreanu”, Bucharest). *Russia:* Vladimir Neroev, Svetlana Saakyan (Moscow Helmholtz Research Institute of Eye Diseases, Moscow), Vladimir Polyakov, Tatiana L Ushakova (Head and Neck Tumors Department, SRI of Pediatric Oncology and Hematology of N.N. Blokhin National Medical Research Center of Oncology of Russian Federation, and Medical Academy of Postgraduate Education, Moscow), Vera A Yarovaya, Andrey A Yarovoy (S.Fyodorov Eye Microsurgery Federal State Institution, Moscow). *Rwanda:* Tuyisabe Theophile (Kabgayi Eye Unit, Gitarama), *Saudi Arabia:* Saleh Al Mesfer, Azza Maktabi (King Khalid eye specialist hospital, Riyadh), Saad A Al-Dahmash, Hind M Alkatan (College of Medicine, King Saud University, Riyadh). *Senegal:* Claude Moreira (Service d’oncologie pédiatrique de l’hôpital Aristide le Dantec, Dakar), Paule Aïda Ndoye Roth (Cheikh Anta DIOP University of Dakar, Le Dantec Hospital, Dakar). *Serbia:* Vesna R Ilic, Marina Nikitovic (Institute for Oncology and Radiology, Belgrade), Slobodanka Latinović (Clinical Center Of Vojvodina – University Eye Clinic, Eye Research Foundation Vidar – Latinović, Novi Sad). *Singapore:* BoonLong Quah, Deborah Tan (Singapore National Eye Centre and KK Women’s and Children’s Hospital in Singapore). *Slovakia:* Stanislava Hederova, Kristina Husakova (University Childrens’ Hospital, Bratislava). *Slovenia:* Alenka Lavric Groznik, Manca Tekavcic Pompe (Univ. Medical Centre Ljubljana, Univ.Eye Hospital Ljubljana, Ljubljana). *South Africa:* Alan Davidson (Red Cross Children’s War Memorial Hospital and the University of Cape Town, Cape Town), Magritha Du Bruyn (University of KwaZulu-Natal, Durban), Johannes Du Plessis, David K Stones (Department of Paediatrics and Child Health, University of the Free Sate, Bloemfontein), Jennifer A Geel, Khumo H Myezo (Division of Paediatric Haematology-Oncology, Charlotte Mexeke Johannesburg Academic Hospital, Faculty of Health Sciences, School of Clinical Medicine, University of the Witwatersrand, Johannesburg), Mariana Kruger (Department of Paediatrics and Child Health, Faculty of Medicine and Health Sciences, Stellenbosch University, Stellenbosch), Ismail Mayet, Gita Naidu, Natasha Naidu (University of the Witwatersrand, Johannesburg), Hamzah Mustak (Division of Ophthalmology, University of Cape Town, Cape Town), David Reynders (University of Pretoria, Pretoria), Julie Wetter (Department of Radiation Oncology, University of Cape Town, Groote Schuur Hospital, Cape Town). *Spain:* Silvia Alarcón Portabella, Nieves Martín-Begue, Charlotte Wolley Dod (Department of Pediatric Ophthalmology, Hospital Vall d’Hebron, Barcelona), Julia Balaguer, Honorio Barranco (Pediatric Oncology Unit, Hospital Universitario y Politécnico La Fe, Valencia), Jaume Català-Mora, Guillermo L Chantada, Maria G Correa Llano (Hospital Sant Joan de Déu, Barcelona), Ana Fernández-Teijeiro, David García Aldana (Hospital Universitario Virgen Macarena, Sevilla), Jesús Peralta Calvo, Sonsoles San Román Pacheco (Pediatric Hemato-Oncology, Hospital Universitario Infantil La Paz, Madrid). *Sri Lanka:* D Sanjeeva Gunasekera (National Cancer Institute, Maharagama). *Sudan:* Moawia MA Elhassan (Oncology Department, National Cancer Institute, University of Gezira, Wadi Madani), Ahmed A Mohamedani (Pathology Department, Faculty of Medicine, University of Gezira, Wadi Madani). *Sweden:* Charlotta All-Eriksson, Katarina Bartuma (St Erik Eye Hospital, Stockholm). *Switzerland:* Maja Beck Popovic (Unit of Pediatric Hematology-Oncology, University Hospital CHUV, Lausanne), Francis L Munier (Jules-Gonin Eye Hospital, Fondation Asile de Aveugles, University of Lausanne, Lausanne). *Taiwan:* Chun-Hsiu Liu (Chang Gung Memorial Hospital, Taipei). *Tanzania:* Faraja S Chiwanga, Alice Kyara (Muhimbili National Hospital, Dar es Salaam), Furahini G Mndeme, Mchikirwa S Msina (Kilimanjaro Christian Medical Centre, Moshi), Trish A Scanlan (Muhimbili National Hospital, Dar es Salaam). *Thailand:* La-ongsri Atchaneeyasakul, Jassada Buaboonnam (Siriraj Hospital Mahidol University, Bangkok), Wantanee Dangboon, Penny Singha (Department of Ophthalmology, Songklanagarind Hospital, Prince of a Songkla university, Songkla), Suradej Hongeng (Department of pediatrics Faculty of medicine Ramathibodi Hospital, Mahidol University, Bangkok), Kittisak Kulvichit (Vitreo-Retina Research Unit, Department of Ophthalmology, Chulalongkorn University, Bangkok), Duangnate Rojanaporn (Department of Ophthalmology, Faculty of Medicine, Ramathibodi Hospital, Mahidol University, Bangkok), Supawan Surukrattanaskul, Nutsuchar Wangtiraumnuay (Queen Sirikit National Institute of Child Health, Bangkok), Atchareeya Wiwatwongwana, Damrong Wiwatwongwana (Department of Ophthalmology, Chiang Mai University, Chiang Mai), Phanthipha Wongwai (Department of ophthalmology, Faculty of Medicine, Khon Kaen University, Khon Kaen). *Timor-Leste:* Manoj K Sharma (East Timor Eye Program, Dili). *Togo:* Koffi M Guedenon (Département de Pédiatrie, CHU Sylvanus Olympio, Université de Lomé, Lomé). *Tunisia:* Hédi Bouguila (Institut Hédi-Raïs d’Ophtalmologie de Tunis, Faculté de Médecine de Tunis, Université Tunis El Manar, Tunis). *Turkey:* Hatice T Atalay, Murat Hasanreisoglu (Gazi University School of Medicine, Department of Ophthalmology, Ankara), Eda Ataseven, Mehmet Kantar (Ege University, School of Medicine, Division of Pediatric Oncology, Izmir), Ahmet K Gündüz (Department of Ophthalmology, Ankara University Faculty of Medicine, Ankara), Rejin Kebudi (Istanbul University, Oncology Institute, Pediatric Hematology-Oncology, Istanbul), Hayyam Kiratli, Irem Ko~ (Ocular Oncology Service, Hacettepe University School of Medicine, Ankara), Samuray Tuncer (Istanbul University, Faculty of Medicine, Department of Ophthalmology, Ocular Oncology Service, Istanbul), Emel Unal (Ankara University Department of Pediatrics, Division of Pediatric Hematology-Oncology, Ankara). *Uganda:* Abubakar Kalinaki (Makerere University College of Health Sciences, Department of Ophthalmology, Kamplala), Marchelo Matua, Keith Waddell (Ruharo Eye Hospital, Mbarara), Anne A Musika, Grace Ssali (Mulago National Referral and Teaching Hospital, Kamplala). *UK:* Lamis Al Harby, M. Ashwin Reddy (The Royal London Hospital, Barts Health NHS Trust, QMUL, London and Moorfields Eye Hospital NHS Foundation Trust, London), Nicholas J Astbury, Covadonga Bascaran, Richard Bowman, Matthew J Burton, Ido Didi Fabian, Allen Foster, Marcia Zondervan (International Centre for Eye Health, London School of Hygiene & Tropical Medicine, London), Richard Bowman (Ophthalmology Department, Great Ormond Street Children’s Hospital, London), Mandeep S Sagoo (NIHR Biomedical Research Centre for Ophthalmology at Moorfields Eye Hospital and UCL Institute of Ophthalmology and London Retinoblastoma Service, Royal London Hospital, London). *Ukraine:* Nadia Bobrova, Tetyana Sorochynska (The Filatov Institute of Eye diseases and Tissue Therapy, Odessa), Lesia Lysytsia (The Okhmatdyt National Children’s Hospital, Kiev). *Uruguay:* Luis Castillo (Hospital Pereira Rossell, Montevideo). *USA:* Armin R Afshar (University of California, San Francisco, CA), Jesse L Berry, Jonathan W Kim, Jasmeen K Randhawa (Children’s Hospital Los Angeles, Keck School of Medicine, University of Southern California, Los Angeles, CA), Elaine Binkley, H C Boldt, Scott A Larson (University of Iowa Department of Ophthalmology, Iowa City, IA), Rachel C Brennan (St. Jude Children’s Research Hospital, Department of Oncology, Solid Tumor Division, Memphis, TN), Arthika Chandramohan, Andrew W Stacey (Department of Ophthalmology, University of Washington, and Seattle Children’s Hospital, Seattle, WA), Timothy W Corson, David A Plager (Indiana University Medical Center, Indianapolis, IN), Jacquelyn M Davanzo, Arun D Singh (Cole Eye Institute, Cleveland Clinic, Cleveland, OH), Hakan Demirci (Department of Ophthalmology and Visual Science, Kellogg Eye Center, University of Michigan, Ann Arbor, MI), Connor Ericksen, George N Magrath (Storm Eye Institute, Medical University of South Carolina, Charleston, SC), Aaron S Gold, Timothy G Murray (Miami Ocular Oncology and Retina, Miami, FL), Efren Gonzalez, Ankoor S Shah (Department of Ophthalmology, Boston Children’s Hospital and Harvard Medical School, Boston, MA), Eric D Hansen, M Elizabeth Hartnett (John A. Moran Eye Center, University of Utah, Salt Lake City, UT), J William Harbour (University of Texas Southwestern Medical Center, Dallas, TX), G. Baker Hubbard, Ogul E Uner (The Emory Eye Center, Atlanta, GA), Kelly D Laurenti, Marilyn B Mets (Ann & Robert H. Lurie Children’s Hospital of Chicago, Division of Ophthalmology, Northwestern University, Feinberg School of Medicine, Chicago, IL), Amy A Leverant, Aparna Ramasubramanian (Phoenix Children’s Hospital, Phoenix, AZ), Sandra Luna-Fineman (Hematology/Oncology/SCT, Center for Global Health, Children’s Hospital Colorado, University of Colorado, Aurora, CO), Audra Miller, Alison H Skalet (Casey Eye Institute, Oregon Health & Science University, Portland, OR), Prithvi Mruthyunjaya, Muhammad Hassan (Byers Eye Institute, Stanford University, Stanford, CA), Scott CN Oliver (Sue Anschutz-Rogers Eye Center at the University of Colorado School of Medicine, Aurora, CO), Carol L Shields, Antonio Yaghy (Ocular Oncology Service, Wills Eye Hospital, Thomas Jefferson University, Philadelphia, PA), Erin D Stahl (Children’s Mercy Hospital, Kansas City, MO), Matthew W Wilson (Department of Surgery, St Jude Children’s Research Hospital, Memphis, TN), Victor M Villegas (Department of Ophthalmology, University of Puerto Rico, San Juan, PR). *Uzbekistan:* Ziyavuddin Islamov, Rustam H Usmanov (National Cancer Center of Uzbekistan, Tashkent). *Venezuela:* Jaime Graells, Livia Romero (Unidad de Oncologia Ocular Hospital Oncologico Luis Razzetti, Caracas). *Vietnam:* Chau TM Pham, Doan L Trang (Vietnam National Institute of Ophthalmology, Ha Noi). *Yemen:* Hamoud HY Al-Hussaini, Abdullah Dahan M Thawaba (Pediatric Oncology Department, National Oncology Center, Sana’a). *Zambia:* Kangwa I M Muma (Ministry of Health, Lusaka), Mutale Nyaywa (Arthur Davison Children’s Hospital, Ndola).
