# Supplementary material for: The Global Retinoblastoma Outcome Study: a prospective, cluster-based analysis of 4064 patients from 149 countries
Source: Lancet Glob Health. Author manuscript; Available in PMC 2022 Aug 23. (PMC9397647; doi:10.1016/S2214-109X(22)00250-9)
Supplement: supplemental [file NIHMS1830257-supplement-supplemental.pdf]

THE LANCET
Global Health

## Supplementary appendix

This appendix formed part of the original submission and has been peer reviewed. We post it as supplied by the authors.

Supplement to: The Global Retinoblastoma Study Group. The Global Retinoblastoma Outcome Study: a prospective, cluster-based analysis of 4064 patients from 149 countries. *Lancet Glob Health* 2022; **10:** e1128–40.

# Relationship between age at diagnosis and hazard

1. Age and survival
   a. Preliminary analysis:
   The linearity of the relationship of log hazard to age at diagnosis was assessed using a pspline (with either 2 or 3 degrees of freedom) for age and all the other specified predictors (income group, laterality, tumor stage, sex, family history of retinoblastoma and hereditary status). The fits were very similar. For age with the 2 df fit, the non-linear component reached a p-value of 4.5*10 to the power -13.

**eFigure 1.**

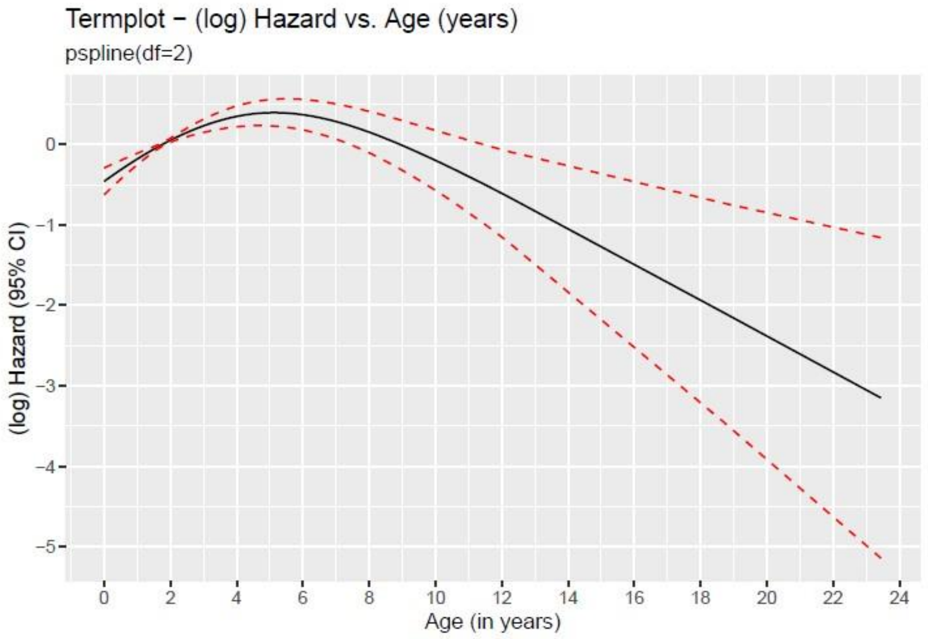


   b. Examples of the relationship between age and hazard in our final model:
      i. For two subjects both less than 3 years, they are affected only by the first term (i.e. age), with coefficient 0.027. If they differ in age by one year, the difference in log hazard is 0.027*12 = 0.324. The hazard ratio (HR) is 1.38.
      ii. Two subjects, one less than 3 years, the other above 3 years, for example 30 and 42 months old. For both subjects, the first term plays in just as above. For the first subject, the second term (age>3 years) = 0. For the second subject, the second term = 6. The difference in log hazard is 0.027*12 - 0.028*6 = 0.155. The HR is 1.17.
      iii. Two subjects both older than 3 years but less than 7 years, and one year apart in age. The difference in log hazard is 0.027*12 - 0.028*12 = -0.013 (i.e. the older subject now has very slightly lower log hazard than the younger one, but the difference is small, as these subjects are "on the plateau"). The HR is 0.99.
   c. **eTable 1** shows the calculated HR (and lower and upper 95% confidence interval, CI) in 6 months steps, compared to age = 12 months (HR=1), from our final model. Greener – larger numbers, redder – smaller numbers.

**eTable 1**

| Age (months) | Hazard Ratio | CI Lower | CI upper | |
|---|---|---|---|---|
| 6 | 0.850912708 | 0.798350929 | 0.906935046 | |
| 12 | 1 | 1 | 1 | |
| 18 | 1.17520868 | 1.102614795 | 1.252581996 | |
| 24 | 1.381115441 | 1.215759386 | 1.568961656 | |
| 30 | 1.623098853 | 1.340514286 | 1.965253122 | |
| 36 | 1.90747986 | 1.478070885 | 2.461640678 | |
| 42 | 1.894596549 | 1.489535469 | 2.409809068 | |
| 48 | 1.881800252 | 1.483431709 | 2.387148776 | |
| 54 | 1.869090383 | 1.459415111 | 2.393766404 | |
| 60 | 1.856466358 | 1.420203148 | 2.426742502 | |
| 66 | 1.843927597 | 1.370118823 | 2.481586944 | |
| 72 | 1.831473524 | 1.313372096 | 2.553956552 | |
| 78 | 1.819103567 | 1.253232781 | 2.640481352 | |
| 84 | 1.806817158 | 1.191964652 | 2.738829742 | |
| 90 | 1.585047386 | 1.121141621 | 2.24090799 | ↑ significant |
| 96 | 1.390497763 | 0.996981728 | 1.939337478 | ↓ non significant |
| 102 | 1.219827272 | 0.833140411 | 1.785987758 | |
| 108 | 1.070104975 | 0.666661329 | 1.71770074 | |
| 114 | 0.938759678 | 0.521115496 | 1.691121721 | |
| 120 | 0.823535778 | 0.402416118 | 1.685347947 | |
| 126 | 0.722454526 | 0.308674444 | 1.690909477 | |
| 132 | 0.633780044 | 0.235828472 | 1.70325975 | |

2. Age and globe salvage - Preliminary analysis:

   A similar analysis as for “age and survival” was performed. For age, the non-linear component reached a p-value of 1.1e$^{-5}$.

**eFigure 2**

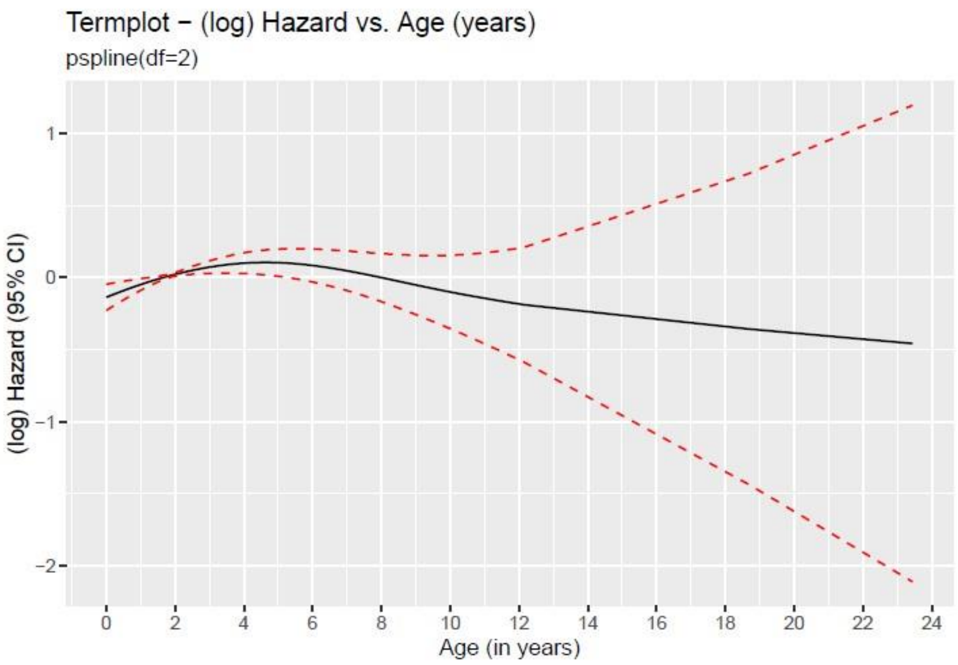

# Clinical characteristics at the time of diagnosis

| eTable 2. Clinical characteristics at the time of diagnosis in 4064 new patients with retinoblastoma in 149 countries in 2017. | | | | | |
|---|---|---|---|---|---|
| | **National income level** | | | | |
| | **No. (% within the national income level) [% within the evaluated parameter]** | | | | |
| **Parameter** | **Low** | **Lower-middle** | **Upper-middle** | **High** | **Total** |
| **Age at diagnosis** | | | | | |
| Median (IQR), mo | 30.0 (18.2 - 46.1) | 24.4 (12.2 - 37.7) | 20.9 (10.0 - 34.3) | 13.6 (6.0 - 27.1) | 23.2 (11.0 - 36.5) |
| Total, No. (%)[a] | 469/480 (97.7) | 1751/1791 (97.8) | 1120/1151 (97.3) | 635/642 (98.9) | 3975/4064 (97.8) |
| **Laterality[b]** | | | | | |
| Unilateral at presentation | 358 (74.6) [12.7] | 1221 (68.2) [43.5] | 815 (70.8) [29.0] | 415 (64.6) [14.8] | 2809 (69.1) |
| Bilateral at presentation | 122 (25.4) [9.7] | 570 (31.8) [45.4] | 336 (29.2) [26.8] | 227 (35.4) [18.1] | 1255 (30.9) |
| **Sex[b]** | | | | | |
| Female | 223 (46.5) [12.2] | 798 (44.6) [43.7] | 527 (45.8) [28.8] | 279 (43.5) [15.3] | 1827 (45.0) |
| Male | 257 (53.5) [11.5] | 993 (55.4) [44.4] | 624 (54.2) [27.9] | 363 (56.5) [16.2] | 2237 (55.0) |
| **Family history of retinoblastoma** | | | | | |
| Yes | 15 (3.4) [7.7] | 73 (4.2) [37.6] | 51 (4.5) [26.3] | 55 (8.6) [28.4] | 194 (4.9) |
| No | 427 (96.6) [11.3] | 1661 (95.8) [44.1] | 1093 (95.5) [29.0] | 583 (91.4) [15.5] | 3764 (95.1) |
| Total, No. (%)[a] | 442/480 (92.1) | 1734/1791 (96.8) | 1144/1151 (99.4) | 638/642 (99.4) | 3958/4064 (97.4) |
| **Clinical Tumor, Node, Metastasis, Heredity 8th Edition Staging** | | | | | |
| **Primary tumor** | | | | | |
| cT1 | 4 (0.9) [1.6] | 87 (5.0) [33.9] | 67 (6.5) [26.1] | 99 (15.6) [38.5] | 257 (6.7) |
| cT2 | 59 (12.9) [5.0] | 364 (20.9) [31.0] | 436 (42.5) [37.1] | 316 (49.7) [26.9] | 1175 (30.5) |
| cT3 | 198 (43.3) [10.9] | 943 (54.3) [51.7] | 469 (45.7) [25.7] | 214 (33.6) [11.7] | 1824 (47.3) |
| cT4 | 196 (42.9) [32.8] | 342 (19.7) [57.2] | 55 (5.4) [9.2] | 5 (0.8) [0.8] | 598 (15.5) |
| Retinoma | 0 | 2 (0.1) [50.0] | 0 | 2 (0.3) [50.0] | 4 (0.1) |
| Total, No. (%)[a] | 457/480 (95.2) | 1738/1791 (97.0) | 1027/1151 (89.2) | 636/642 (99.1) | 3858/4064 (94.9) |
| **Regional lymph node** | | | | | |
| NX | 78 (16.9) [11.6] | 263 (15.0) [39.0] | 226 (19.9) [33.5] | 108 (16.9) [16.0] | 675 (16.9) |
| N0 | 340 (73.8) [10.7] | 1409 (80.3) [44.5] | 884 (78.0) [27.9] | 531 (83.1) [16.8] | 3164 (79.3) |
| N1 | 43 (9.3) [28.9] | 82 (4.7) [55.0] | 24 (2.1) [16.1] | 0 | 149 (3.7) |
| Total, No. (%)[a] | 461/480 (96.0) | 1754/1791 (97.9) | 1134/1151 (98.5) | 639/642 (99.5) | 3988/4064 (98.1) |
| **Distant metastasis** | | | | | |
| M0 | 368 (81.4) [10.0] | 1597 (91.1) [43.4] | 1082 (95.2) [29.4] | 636 (99.5) [17.3] | 3683 (92.5) |
| cM1 | 56 (12.4) [28.4] | 104 (5.9) [52.8] | 36 (3.2) [18.3] | 1 (0.2) [0.5] | 197 (4.9) |
| pM1 | 28 (6.2) [27.7] | 52 (3.0) [51.5] | 19 (1.7) [18.8] | 2 (0.3) [2.0] | 101 (2.5) |
| Total, No. (%)[a] | 452/480 (94.2) | 1753/1791 (97.9) | 1137/1151 (98.8) | 639/642 (99.5) | 3981/4064 (98.0) |
| **Hereditary trait** | | | | | |
| HX | 320 (70.8) [13.6] | 1106 (63.3) [46.9] | 708 (62.4) [30.0] | 225 (35.2) [9.5] | 2359 (59.4) |
| H0 | 0 | 42 (2.4) [17.6] | 55 (4.8) [23.1] | 141 (22.0) [59.2] | 238 (6.0) |
| H1 | 132 (29.2) [9.6] | 598 (34.2) [43.5] | 372 (32.8) [27.0] | 274 (42.8) [19.9] | 1376 (34.6) |
| Total, No. (%)[a] | 452/480 (94.2) | 1746/1791 (97.5) | 1135/1151 (98.6) | 640/642 (99.7) | 3973/4064 (97.8) |

[a]The number of individuals for whom data were available.
[b]Inclusion criterion: 100% reporting.
Abbreviations: IQR - interquartile range.

# Treatments

| eTable 3. Diagnostic and treatment modalities available for 4064 new patients with retinoblastoma diagnosed in 149 countries in 2017 | | | | | |
|---|---|---|---|---|---|
| **Parameter** | **National income level (n (%))** | | | | |
| | **Low** (n=480) | **Lower-middle** (n=1,791) | **Upper-middle** (n=1,151) | **High** (n=642) | **Total** (n=4,064) |
| Genetic tests | 5 (1.0) | 369 (20.6) | 505 (43.9) | 586 (91.3) | 1,465 (36.0) |
| CT | 231 (48.1) | 1217 (68.0) | 860 (74.7) | 388 (60.4) | 2696 (66.3) |
| MRI | 164 (34.2) | 1317 (73.5) | 1082 (94.0) | 638 (99.4) | 3201 (78.8) |
| CT + MRI | 76 (15.8) | 805 (44.9) | 837 (72.7) | 384 (59.8) | 2,102 (51.7) |
| Pathology | 462 (96.3) | 1,759 (98.2) | 1,128 (98.0) | 637 (99.2) | 3986 (98.1) |
| Laser therapy | 244 (50.8) | 1,451 (81.0) | 995 (86.4) | 634 (98.8) | 3,324 (81.8) |
| Cryotherapy | 157 (32.7) | 1,243 (69.4) | 939 (81.6) | 634 (98.8) | 2793 (73.2) |
| Enucleation | Available for all children | | | | |
| Intravenous chemotherapy | 462 (96.3) | 1,787 (99.8) | 1,124 (97.7) | 641 (99.8) | 4,014 (98.8) |
| Intra-ophthalmic artery chemotherapy | 9 (1.9) | 550 (30.7) | 811 (70.5) | 538 (83.8) | 1908 (46.9) |
| Intravitreal chemotherapy | 148 (30.8) | 1,055 (58.9) | 980 (85.1) | 631 (98.3) | 2,814 (69.2) |
| Plaque brachytherapy | 5 (1.0) | 410 (22.9) | 231 (20.1) | 483 (75.2) | 1,129 (27.8) |
| External beam radiotherapy | 194 (40.4) | 1,403 (78.3) | 793 (68.9) | 524 (81.6) | 2914 (71.7) |

| eTable 4. Primary and additional treatments given to 4064 new patients with retinoblastoma diagnosed in 149 countries in 2017 | | | | | |
|---|---|---|---|---|---|
| | **National income level** | | | | |
| **Treatment**[a] | **Low** (n=474) | **Lower-middle** (n=1783) | **Upper-middle** (n=1148) | **High** (n=638) | **Total** (n=4043) |
| **Primary treatment for patient** No. (%)[b] | | | | | |
| Intravenous chemotherapy | 276 (58.2) | 880 (49.4) | 502 (43.7) | 279 (43.7) | 1937 (47.9) |
| Intra-ophthalmic artery chemotherapy | 0 | 17 (1.0) | 181 (15.8) | 106 (16.8) | 304 (7.5) |
| Enucleation[c] | 129 (27.2) | 730 (40.9) | 454 (39.5) | 258 (40.4) | 1571 (38.9) |
| Exenteration[c] | 13 (2.7) | 40 (2.2) | 1 (0.1) | 0 | 54 (1.3) |
| Focal laser or cryotherapy | 18 (3.8) | 110 (6.2) | 39 (3.4) | 54 (8.5) | 221 (5.5) |
| Plaque brachytherapy | 0 | 0 | 0 | 3 (0.5) | 3 (0.1) |
| Vitrectomy | 0 | 0 | 2 (0.2) | 1 (0.2) | 3 (0.1) |
| Palliative therapy | 25 (5.3) | 23 (1.3) | 0 | 0 | 48 (1.2) |
| Observation | 0 | 2 (0.1) | 0 | 2 (0.3) | 4 (0.1) |
| Primary treatment refusal | 34 (7.2) | 174 (9.8) | 37 (3.2) | 10 (1.6) | 255 (6.3) |
| **Additional treatments for patient** No. (%)[d] | | | | | |
| Intravenous chemotherapy | 160 (33.8) | 634 (35.6) | 392 (34.1) | 125 (19.6) | 1311 (32.4) |
| Intra-ophthalmic artery chemotherapy | 2 (0.4) | 45 (2.5) | 226 (19.7) | 148 (23.2) | 421 (10.4) |
| Enucleation[b] | 201 (42.2) | 444 (24.9) | 252 (22.0) | 115 (18.0) | 1012 (25.0) |
| Exenteration[b] | 10 (2.1) | 20 (1.1) | 8 (0.7) | 2 (0.3) | 40 (1.0) |
| Focal laser or cryotherapy | 52 (11.0) | 303 (17.0) | 346 (30.1) | 294 (46.1) | 995 (24.6) |

| | | | | | |
|---|---|---|---|---|---|
| Intravitreal chemotherapy | 10 (2.1) | 87 (4.9) | 145 (12.6) | 101 (15.8) | 343 (8.5) |
| Teletherapy[e] | 20 (4.2) | 112 (6.3) | 49 (4.3) | 11 (1.7) | 192 (4.7) |
| Plaque brachytherapy | 0 | 22 (1.2) | 34 (3.0) | 52 (8.2) | 108 (2.7) |
| Vitrectomy | 0 | 14 (0.8) | 21 (1.8) | 5 (0.8) | 40 (1.0) |
| Palliative therapy | 2 (0.4) | 16 (0.9) | 0 | 0 | 18 (0.4) |
| Treatment refusal after primary treatment | 27 (5.7) | 88 (4.9) | 38 (3.3) | 2 (0.3) | 155 (3.8) |

[a]Per patient.
[b]First and main treatment. In case of combined enucleation/exenteration and chemotherapy, both were counted as primary treatment. In case of enucleation/exenteration/chemotherapy and other listed therapies, the other therapies were not counted as primary treatment.
[c]Primary and secondary enucleation and exenteration numbers do not match the total number in the text, because in this table patients who underwent bilateral surgery could be counted once or twice, depending on the clinical scenario, whereas in the text a patient who underwent enucleation in one or both eyes is counted once.
[d]Additiional treatment for tumor relapse or new tumors. Additional treatments not listed separately: intravitreal anti vascular endothelial growth factor antibody (n=5), cataract surgery (n=2), episcleral topotecan implant (n=1), intracameral chemotherapy (n=1), intrathecal chemotherapy (n=10), intraorbital chemotherapy (n=28), scleral buckle for retinal detachment (n=2), sphincterotomy (n=1), and sub-Tenon chemotherapy (n=6).
[e]External beam radiotherapy, proton beam radiotherapy, or gamma knife radiosurgery.

# Survival rate by national income level and by clinical stage

**eTable 5. 1, 2, and 3-year survival rate by national income level and by clinical stage in 4064 new patients with retinoblastoma diagnosed in 149 countries in 2017.**

| | Time (mo) | Survival Rate (%) | SE (%) | Lower 95% CI | Upper 95% CI |
|---|---|---|---|---|---|
| **National Income Level** | | | | | |
| **Low** | **12** | 74.4 | 2.1 | 70.3 | 78.8 |
| | **24** | 62.5 | 2.5 | 57.8 | 67.6 |
| | **36** | 57.3 | 2.8 | 52.1 | 63.0 |
| **Lower middle** | **12** | 88.4 | 0.8 | 86.8 | 90.0 |
| | **24** | 82.8 | 1.0 | 80.9 | 84.7 |
| | **36** | 80.3 | 1.0 | 78.3 | 82.3 |
| **Upper middle** | **12** | 95.1 | 0.7 | 93.8 | 96.4 |
| | **24** | 91.9 | 0.8 | 90.3 | 93.6 |
| | **36** | 91.2 | 0.9 | 89.5 | 93.0 |
| **High** | **12** | 99.8 | 0.2 | 99.5 | 100.0 |
| | **24** | 99.7 | 0.2 | 99.2 | 100.0 |
| | **36** | 99.5 | 0.3 | 98.8 | 100.0 |
| **Clinical Stage** | | | | | |
| **cT1** | **12** | 97.7 | 0.9 | 95.9 | 99.6 |
| | **24** | 97.1 | 1.1 | 95.1 | 99.2 |
| | **36** | 94.4 | 0.7 | 93.0 | 95.8 |
| **cT2** | **12** | 97.6 | 0.5 | 96.7 | 98.5 |
| | **24** | 95.7 | 0.6 | 94.5 | 96.9 |
| | **36** | 94.4 | 0.7 | 93.0 | 95.8 |
| **cT3** | **12** | 95.3 | 0.5 | 94.4 | 96.3 |
| | **24** | 91.3 | 0.7 | 90.0 | 92.7 |
| | **36** | 90.5 | 0.7 | 89.1 | 91.9 |
| **cT4** | **12** | 55.0 | 2.2 | 50.8 | 59.6 |
| | **24** | 39.5 | 2.3 | 35.2 | 44.3 |
| | **36** | 31.9 | 2.4 | 27.6 | 36.9 |

Abbreviations: SE - Standard error, CI – confidence interval.

# Sensitivity analysis for survival

In these sensitivity analyses, we test the effect of weighting, test whether dropping patients with missing values of predictor variables, rather than imputing the missing values, is of significance, test the impact of extending the declaration of missing survival outcomes to participants with follow-up times of 1 month or less, and test whether the relationship of log hazard to age at diagnosis differs by hereditary status.

## Weighted vs. no-weights

**eFigure 3. Whole cohort survival analysis by Kaplan-Meier method: weighted vs. no-weights**

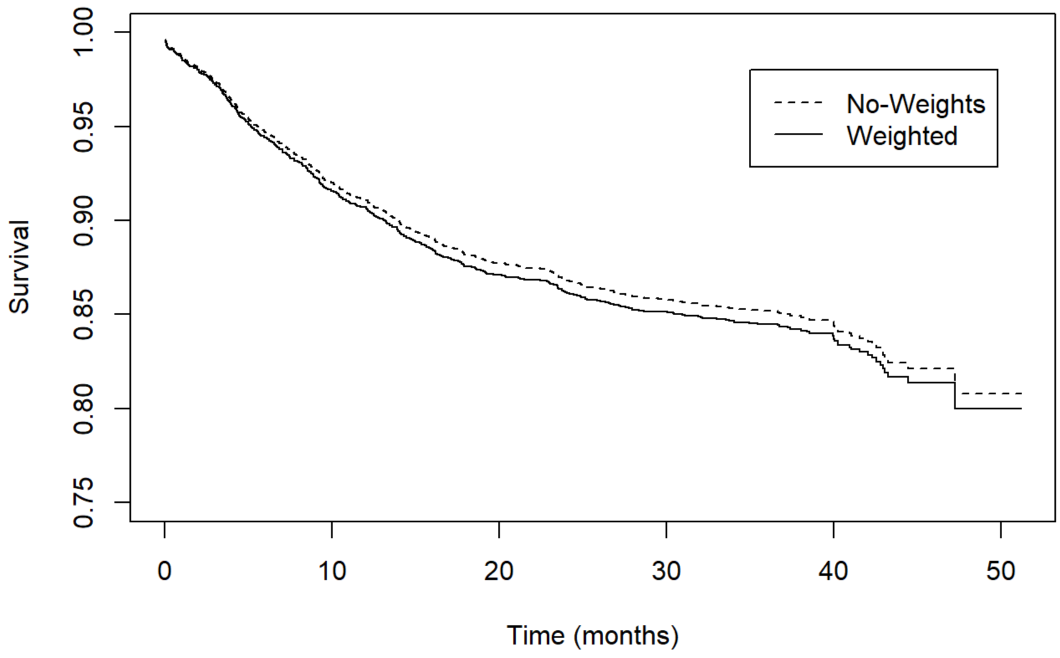


**eFigure 4. Survival Kaplan-Meier model by income group: weighted vs. no-weights**

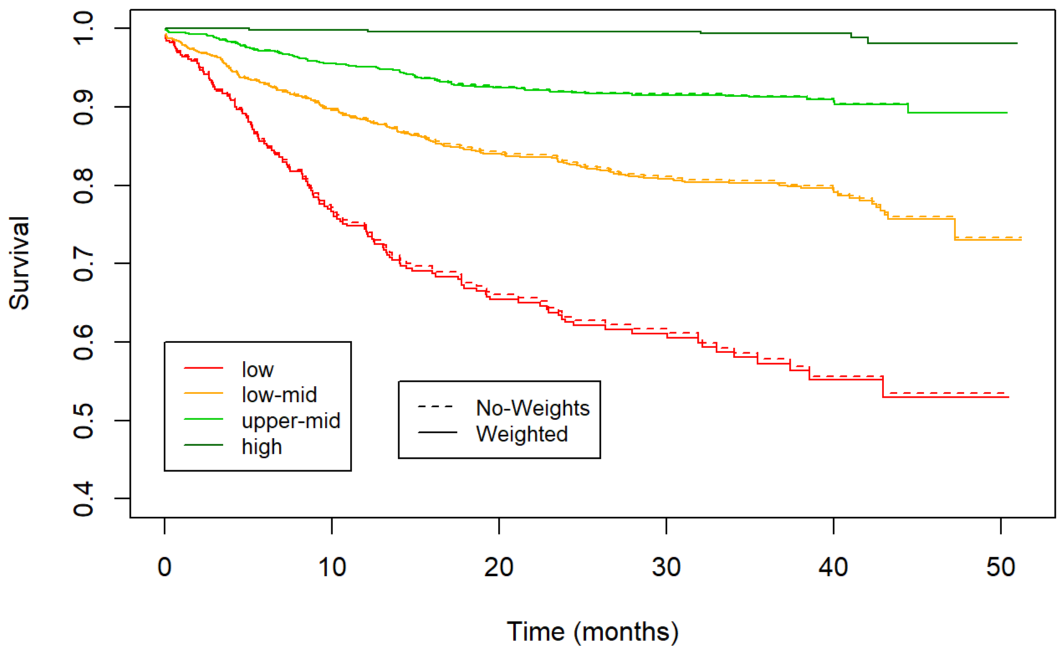

**eTable 6. Non-weighted Cox proportional hazards model for survival in 4064 new patients with retinoblastoma diagnosed in 149 countries in 2017[a].**

| | Coefficient | Robust SE | Z value | P-value (corrected[b]) | Hazard ratio | Lower 95% CI | Upper 95% CI |
|---|---|---|---|---|---|---|---|
| **Country of residence income** | | | | | | | |
| Low | Reference | | | | 1.00 | | |
| Lower-middle | -0.293 | 0.210 | -1.395 | 0.16 (0.99) | 0.75 | 0.49 | 1.13 |
| Upper-middle | -0.603 | 0.263 | -2.292 | 0.022 (0.29) | 0.55 | 0.33 | 0.92 |
| High | -2.787 | 0.654 | -4.258 | <0.001 (<0.001) | 0.06 | 0.02 | 0.22 |
| **Age[c]** | | | | | | | |
| Age HR/mo | 0.027 | 0.006 | 4.862 | <0.001 (<0.001) | 1.03 | 1.02 | 1.04 |
| Age>3yr HR/mo | -0.028 | 0.008 | -3.281 | 0.001 (0.013) | 0.97 | 0.96 | 0.99 |
| Age>7yr HR/mo | -0.022 | 0.016 | -1.367 | 0.17 (0.99) | 0.98 | 0.95 | 1.01 |
| **Bilaterality** | | | | | | | |
| Unilateral | Reference | | | | 1.00 | | |
| Bilateral | 0.451 | 0.565 | 0.798 | 0.42 (0.99) | 1.57 | 0.52 | 4.76 |
| **Primary tumor** | | | | | | | |
| cT1 | Reference | | | | 1.00 | | |
| cT2 | 0.080 | 0.293 | 0.274 | 0.78 (0.99) | 1.08 | 0.61 | 1.9 |
| cT3 | 0.174 | 0.335 | 0.520 | 0.60 (0.99) | 1.19 | 0.62 | 2.2 |
| cT4 | 2.236 | 0.364 | 6.149 | <0.001 (<0.001) | 9.35 | 4.59 | 19.07 |
| **Sex** | | | | | | | |
| Male | Reference | | | | 1.00 | | |
| Female | 0.126 | 0.078 | 1.611 | 0.11 (0.99) | 1.13 | 0.97 | 1.32 |
| **Family history of retinoblastoma** | | | | | | | |
| Yes | Reference | | | | 1.00 | | |
| No | 0.170 | 0.334 | 0.509 | 0.61 (0.99) | 1.19 | 0.62 | 2.28 |
| **Hereditary retinoblastoma** | | | | | | | |
| Non-hereditary | Reference | | | | 1.00 | | |
| Hereditary[d] | -0.233 | 0.582 | -0.400 | 0.69 (0.99) | 0.79 | 0.25 | 2.48 |

[a]N=3564, number of events (i.e. death) = 484. 500 observations deleted due to missing observation time.
[b]Multiplied by 13, according to Bonferroni's model.
[c]Age included in the analysis as a continuous parameter. The relation of log hazard to age at diagnosis is estimated by three coefficients, which point to increasing hazard through age 3, then stable hazard until age 7, and then decreasing hazard at older ages. For further details, see "Relationship between age at time of diagnosis and log hazard" in the **Supplement**.
[d]Hereditary - bilateral/trilateral retinoblastoma, positive family history or positive blood *RB1* mutation (H1 in cTNMH).
Abbreviations: SE - Standard error, CI – confidence interval, yr – years, mo – months.

## Impute vs. drop missing x values

**eFigure 5. Whole cohort survival analysis by Kaplan-Meier model: impute vs. delete cases with missing predictors**

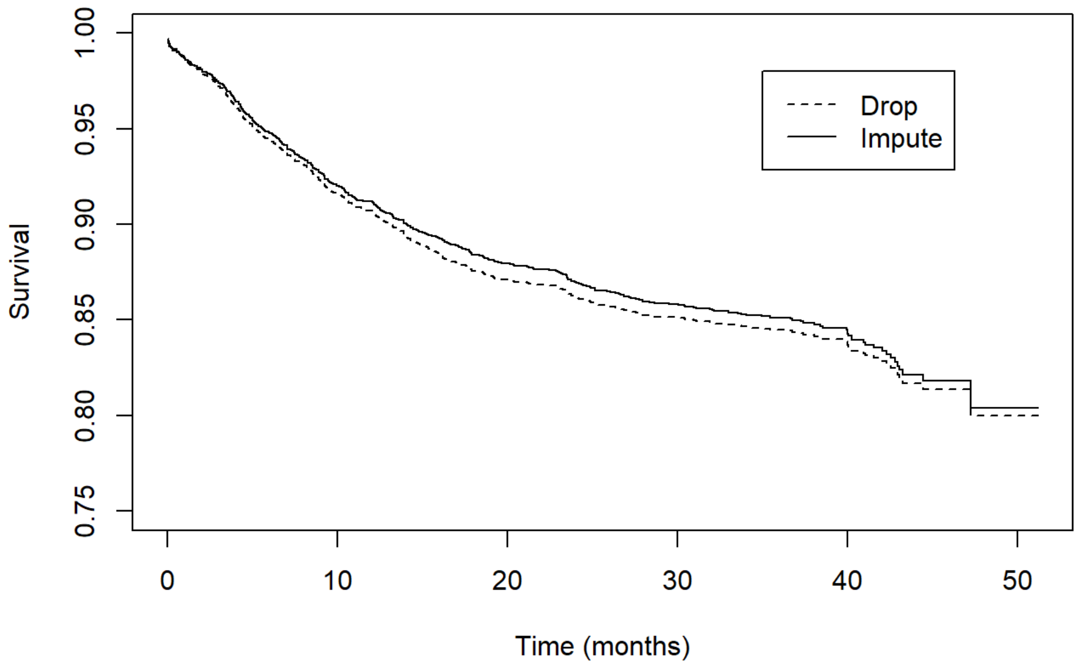


| **eTable 7. Weighted Cox proportional hazard model for survival in 4064 new patients with retinoblastoma diagnosed in 149 countries in 2017[a]: dropped missing x.** | | | | | | | |
|---|---|---|---|---|---|---|---|
| | **Coefficient** | **Robust SE** | **Z value** | **P-value (corrected[b])** | **Hazard ratio** | **Lower 95% CI** | **Upper 95% CI** |
| **Country of residence income** | | | | | | | |
| Low | reference | | | | 1.00 | | |
| Lower-middle | -0.264 | 0.223 | -1.183 | 0.24 (0.99) | 0.77 | 0.50 | 1.19 |
| Upper-middle | -0.462 | 0.284 | -1.625 | 0.10 (0.99) | 0.63 | 0.36 | 1.10 |
| High | -2.671 | 0.661 | -4.042 | <0.001 (<0.001) | 0.07 | 0.02 | 0.25 |
| **Age[c]** | | | | | | | |
| Age HR/mo | 0.026 | 0.006 | 4.445 | <0.001 (<0.001) | 1.03 | 1.01 | 1.04 |
| Age>3yr HR/mo | -0.027 | 0.009 | -3.011 | 0.003 (0.034) | 0.97 | 0.96 | 0.99 |
| Age>7yr HR/mo | -0.021 | 0.018 | -1.221 | 0.22 (0.99) | 0.98 | 0.95 | 1.01 |
| **Bilaterality** | | | | | | | |
| Unilateral | reference | | | | 1.00 | | |
| Bilateral | 0.302 | 0.549 | 0.550 | 0.58 (0.99) | 1.35 | 0.46 | 3.97 |
| **Primary tumor** | | | | | | | |
| cT1 | reference | | | | 1.00 | | |
| cT2 | 0.120 | 0.300 | 0.402 | 0.69 (0.99) | 1.13 | 0.63 | 2.03 |
| cT3 | 0.206 | 0.363 | 0.568 | 0.57 (0.99) | 1.23 | 0.60 | 2.50 |
| cT4 | 2.344 | 0.404 | 5.796 | <0.001 (<0.001) | 10.43 | 4.72 | 23.04 |
| **Sex** | | | | | | | |
| Male | reference | | | | 1.00 | | |
| Female | 0.126 | 0.086 | 1.466 | 0.14 (0.99) | 1.13 | 0.96 | 1.34 |
| **Family history of retinoblastoma** | | | | | | | |
| Yes | reference | | | | 1.00 | | |
| No | 0.233 | 0.324 | 0.720 | 0.47 (0.99) | 1.26 | 0.67 | 2.80 |

| Hereditary retinoblastoma | | | | | | | |
|---|---|---|---|---|---|---|---|
| Non-hereditary | Reference | | | | 1.00 | | |
| Hereditary[d] | -0.072 | 0.562 | -0.127 | 0.90 (0.99) | 0.93 | 0.31 | 2.80 |

[a]N=3303, number of events (i.e. death) = 438. 404 observations deleted due to missing observation time.
[b]Multiplied by 13, according to Bonferroni's model.
[c]Age included in the analysis as a continuous parameter. The relation of log hazard to age at diagnosis is estimated by three coefficients, which point to increasing hazard through age 3, then stable hazard until age 7, and then decreasing hazard at older ages. For examples, see "Relationship between age at time of diagnosis and log hazard" in the **Supplement**.
[d]Hereditary - bilateral/trilateral retinoblastoma, positive family history or positive blood *RB1* mutation (H1 in cTNMH).
Abbreviations: SE - Standard error, CI – confidence interval, yr – years, mo – months.

## Treating no-death with last follow-up≤1 month as missing

**eFigure 6. Whole cohort survival analysis by Kaplan-Meier model: time=0 vs. time≤1 month as no follow-up (NA)**

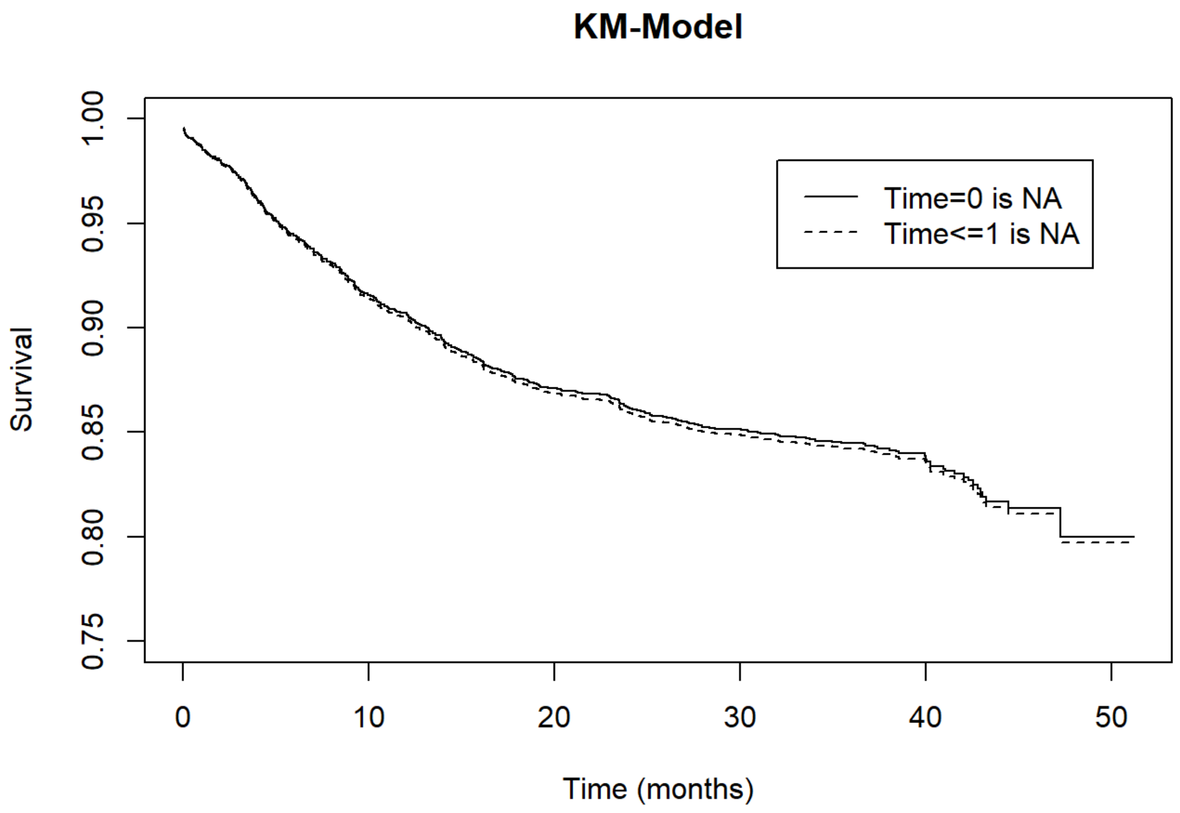


| eTable 8. Weighted Cox proportional hazard model for survival in 4064 new patients with retinoblastoma diagnosed in 149 countries in 2017[a]: time≤1 month treated as no follow-up. | | | | | | | |
|---|---|---|---|---|---|---|---|
| | **Coefficient** | **Robust SE** | **Z value** | **P-value (corrected[b])** | **Hazard ratio** | **Lower 95% CI** | **Upper 95% CI** |
| **Country of residence income** | | | | | | | |
| Low | reference | | | | 1.00 | | |
| Lower-middle | -0.304 | 0.208 | -1.466 | 0.14 (0.99) | 0.74 | 0.49 | 1.11 |
| Upper-middle | -0.629 | 0.260 | -2.418 | 0.016 (0.20) | 0.53 | 0.32 | 0.89 |
| High | -2.825 | 0.651 | -4.339 | <0.001 (<0.001) | 0.06 | 0.02 | 0.21 |

| | | | | | | | |
|---|---|---|---|---|---|---|---|
| **Age**[c] | | | | | | | |
| Age HR/mo | 0.027 | 0.005 | 4.947 | <0.001 (<0.001) | 1.03 | 1.02 | 1.04 |
| Age>3yr HR/mo | -0.028 | 0.008 | -3.379 | 0<0.001 (0.009) | 0.97 | 0.96 | 0.99 |
| Age>7yr HR/mo | -0.020 | 0.015 | -1.290 | 0.197 (0.99) | 0.98 | 0.95 | 1.01 |
| **Bilaterality** | | | | | | | |
| Unilateral | reference | | | | 1.00 | | |
| Bilateral | 0.427 | 0.572 | 0.747 | 0.455 (0.99) | 1.53 | 0.50 | 4.70 |
| **Primary tumor** | | | | | | | |
| cT1 | reference | | | | 1.00 | | |
| cT2 | 0.073 | 0.294 | 0.248 | 0.80 (0.99) | 1.08 | 0.60 | 1.91 |
| cT3 | 0.183 | 0.335 | 0.545 | 0.59 (0.99) | 1.20 | 0.62 | 2.31 |
| cT4 | 2.200 | 0.359 | 6.120 | <0.001 (<0.001) | 9.02 | 4.46 | 18.25 |
| **Sex** | | | | | | | |
| Male | reference | | | | 1.00 | | |
| Female | 0.126 | 0.077 | 1.639 | 0.10 (0.99) | 1.13 | 0.98 | 1.32 |
| **Family history of retinoblastoma** | | | | | | | |
| Yes | reference | | | | 1.00 | | |
| No | 0.188 | 0.337 | 0.558 | 0.58 (0.99) | 1.21 | 0.62 | 2.34 |
| **Hereditary retinoblastoma** | | | | | | | |
| Non-hereditary | Reference | | | | 1.00 | | |
| Hereditary[d] | -0.234 | 0.590 | -0.396 | 0.69 (0.99) | 0.79 | 0.25 | 2.51 |

[a]N=3465, number of events (i.e. death) = 484. 599 observations deleted due to missing observation time.
[b]Multiplied by 13, according to Bonferroni's model.
[c]Age included in the analysis as a continuous parameter. The relation of log hazard to age at diagnosis is estimated by three coefficients, which point to increasing hazard through age 3, then stable hazard until age 7, and then decreasing hazard at older ages. For examples, see "Relationship between age at time of diagnosis and log hazard" in the **Supplement**.
[d]Hereditary - bilateral/trilateral retinoblastoma, positive family history or positive blood *RB1* mutation (H1 in cTNMH).
Abbreviations: SE - Standard error, CI – confidence interval, yr – years, mo – months.

## Analysis of age at diagnosis by hereditary status

**eTable 9. Weighted Cox proportional hazard model for survival in 4064 new patients with retinoblastoma diagnosed in 149 countries in 2017[a]: relation of hazard to age dependent on hereditary status.**

| | **Coefficient** | **Robust SE** | **Z value** | **P-value (corrected[b])** | **Hazard ratio** | **Lower 95% CI** | **Upper 95% CI** |
|---|---|---|---|---|---|---|---|
| **Country of residence income** | | | | | | | |
| Low | reference | | | | 1.00 | | |
| Lower-middle | -0.300 | 0.209 | -1.431 | 0.15 (0.99) | 0.74 | 0.49 | 1.12 |
| Upper-middle | -0.633 | 0.261 | -2.421 | 0.015 (0.20) | 0.53 | 0.32 | 0.89 |
| High | -2.830 | 0.652 | -4.340 | <0.001 (<0.001) | 0.06 | 0.02 | 0.21 |
| **Age**[c] | | | | | | | |
| Age HR/mo | 0.030 | 0.006 | 4.610 | <0.001 (<0.001) | 1.03 | 1.02 | 1.04 |
| Age>3yr HR/mo | -0.030 | 0.009 | -3.480 | <0.001 (0.007) | 0.97 | 0.95 | 0.99 |
| Age>7yr HR/mo | -0.020 | 0.015 | -1.375 | 0.17 (0.99) | 0.98 | 0.95 | 1.01 |
| **Bilaterality** | | | | | | | |
| Unilateral | reference | | | | 1.00 | | |
| Bilateral | 0.406 | 0.569 | 0.713 | 0.48 (0.99) | 1.50 | 0.49 | 4.58 |
| **Primary tumor** | | | | | | | |

| | | | | | | | |
|---|---|---|---|---|---|---|---|
| cT1 | reference | | | | 1.00 | | |
| cT2 | 0.061 | 0.297 | 0.206 | 0.84 (0.99) | 1.06 | 0.59 | 1.90 |
| cT3 | 0.167 | 0.336 | 0.496 | 0.62 (0.99) | 1.18 | 0.61 | 2.28 |
| cT4 | 2.180 | 0.360 | 6.063 | <0.001 (<0.001) | 8.85 | 4.37 | 17.90 |
| **Sex** | | | | | | | |
| Male | reference | | | | 1.00 | | |
| Female | 0.132 | 0.077 | 1.702 | 0.09 (0.99) | 1.14 | 0.98 | 1.33 |
| **Family history of retinoblastoma** | | | | | | | |
| Yes | reference | | | | 1.00 | | |
| No | 0.213 | 0.338 | 0.629 | 0.53 (0.99) | 1.24 | 0.64 | 2.40 |
| **Hereditary retinoblastoma** | | | | | | | |
| Non-hereditary | Reference | | | | 1.00 | | |
| Hereditary[d] | -0.064 | 0.628 | -0.102 | 0.92 (0.99) | 0.94 | 0.27 | 3.21 |
| **Age – hereditary interaction[e]** | | | | | | | |
| Age:hereditary | -0.003 | 0.010 | -0.332 | 0.74 (0.99) | 1.00 | 0.98 | 1.02 |
| Age>3yr:hereditary | -0.014 | 0.020 | -0.694 | 0.49 (0.99) | 0.99 | 0.95 | 1.03 |

[a]N=3564, number of events (i.e. death) = 484. 500 observations deleted due to missing observation time.
[b]Multiplied by 13, according to Bonferroni's model.
[c]Age included in the analysis as a continuous parameter. The relation of log hazard to age at diagnosis is estimated by three coefficients, which point to increasing hazard through age 3, then stable hazard until age 7, and then decreasing hazard at older ages. For examples, see "Relationship between age at time of diagnosis and log hazard" in the **Supplement**.
[d]Hereditary - bilateral/trilateral retinoblastoma, positive family history or positive blood *RB1* mutation (H1 in cTNMH).
[e]Estimation of the difference between hereditary and non-hereditary patients in the relation of log hazard to age. Age>7 years not shown because there were only few patients in this age category.
Abbreviations: SE - Standard error, CI – confidence interval, yr – years, mo – months.

## Survival - Multisample Imputation – Death

**eTable 10. Multisample imputation for survival in 4064 new patients with retinoblastoma diagnosed in 149 countries in 2017.**

| **Parameter** | | **Estimated coefficients** | **Standard errors** |
|---|---|---|---|
| **Age** | Age | 0.026 | 0.006 |
| | Age>3yr | -0.026 | 0.009 |
| | Age>7yr | -0.023 | 0.016 |
| **Country of residence income** | Lower-middle | -0.313 | 0.205 |
| | Upper-middle | -0.674 | 0.269 |
| | High | -2.853 | 0.651 |
| **Laterality** | Bilateral | 0.503 | 0.561 |
| **Primary tumor** | cT2 | 0.036 | 0.322 |
| | cT3 | 0.096 | 0.345 |
| | cT4 | 2.115 | 0.361 |
| **Sex** | Female | 0.127 | 0.078 |
| **Family history of retinoblastoma** | Yes | 0.118 | 0.337 |
| **Hereditary retinoblastoma** | Yes | -0.305 | 0.581 |

# Globe Salvage Analysis by Competing Risks

**eFigure 7. Globe salvage analysis by competing risks.**

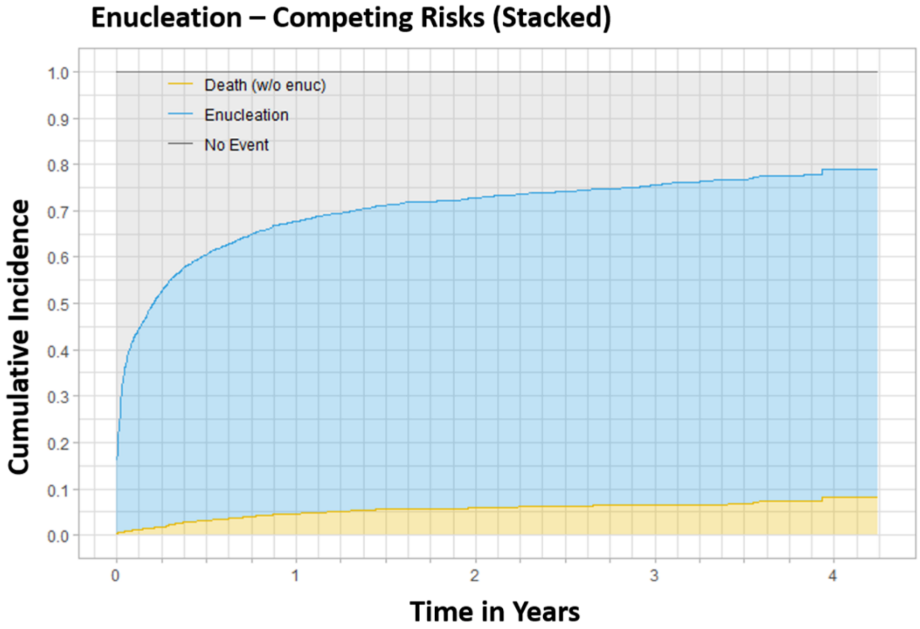


**Number at risk**

4064 1494 1162 1015 930 832 632 285 12

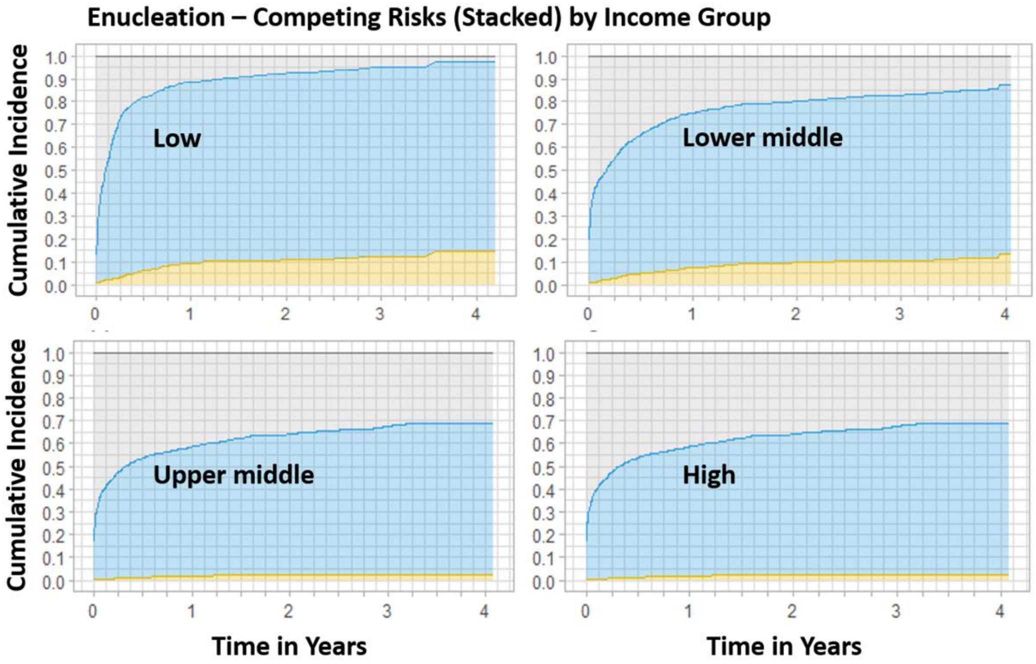


**Upper panel**: Competing risks plot (stacked), whole cohort analysis. **Lower panel**: Competing risks plot (stacked) by income group.

# Sensitivity Analysis for Globe Salvage

In these sensitivity analyses, we test the weighting effect, and test whether dropping patients with missing values of predictor variables, rather than imputing the missing values, is of significance.

**eFigure 8. Competing risks for globe salvage, whole cohort analysis: weighted vs. no-weights**

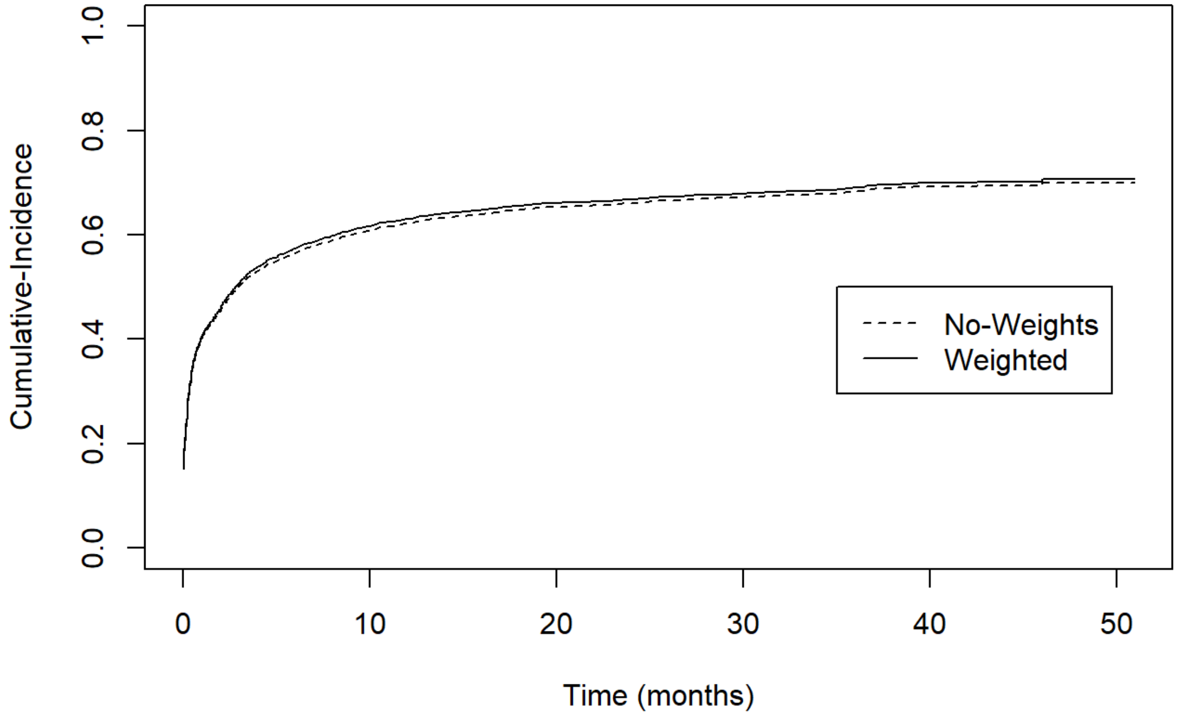


**eFigure 9. Competing risks for globe salvage by income group: weighted vs. no-weights**

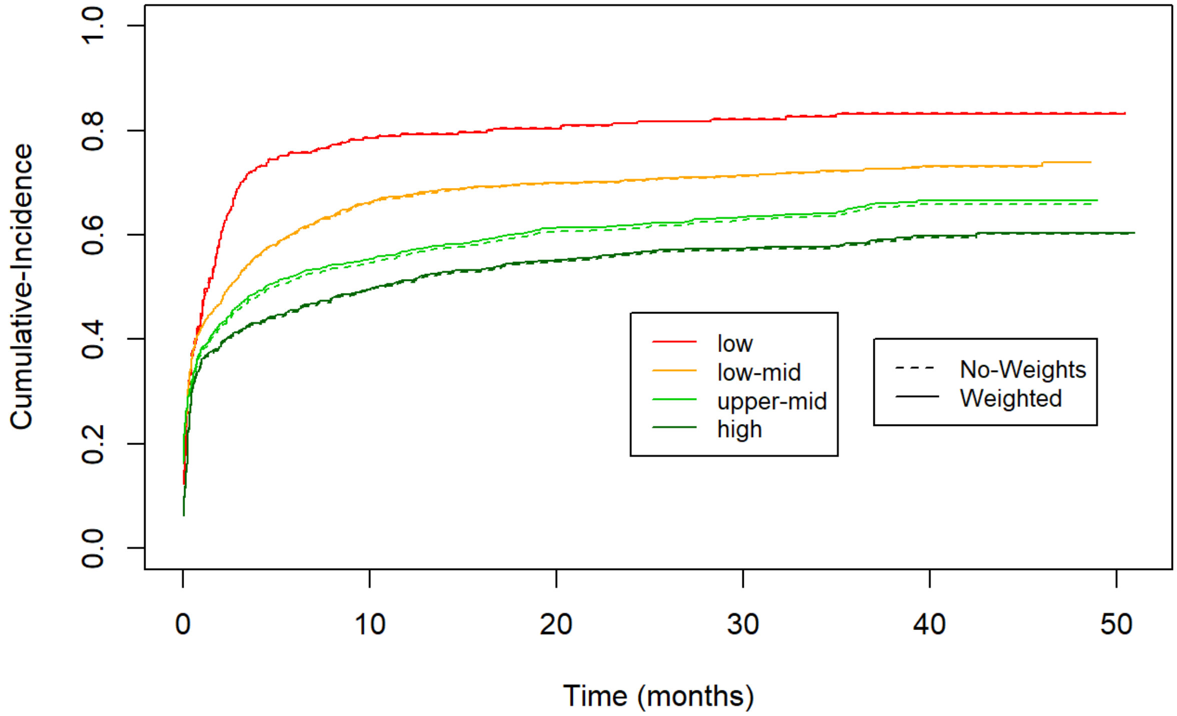

**eTable 11. Non-weighted Cox proportional hazard model for globe salvage in 4064 new patients with retinoblastoma diagnosed in 149 countries in 2017[a].**

| | Coefficient | Robust SE | Z value | P-value[b] | Hazard ratio | Lower 95% CI | Upper 95% CI |
|---|---|---|---|---|---|---|---|
| **Country of residence income** | | | | | | | |
| Low | Reference | | | | 1.00 | | |
| Lower-middle | -0.171 | 0.117 | -1.462 | 0.14 (0.99) | 0.84 | 0.67 | 1.06 |
| Upper-middle | -0.356 | 0.204 | -1.744 | 0.08 (0.99) | 0.70 | 0.47 | 1.05 |
| High | -0.161 | 0.168 | -0.959 | 0.34 (0.99) | 0.85 | 0.61 | 1.18 |
| **Age[c]** | | | | | | | |
| Age HR/mo | 0.007 | 0.002 | 3.466 | <0.001 (0.007) | 1.01 | 1.00 | 1.01 |
| Age>4yr HR/mo | -0.013 | 0.003 | -3.796 | <0.001 (0.002) | 0.99 | 0.98 | 0.99 |
| **Bilaterality** | | | | | | | |
| Unilateral | Reference | | | | 1.00 | | |
| Bilateral | -0.471 | 0.140 | -3.370 | <0.001 (0.001) | 0.62 | 0.47 | 0.82 |
| **Primary tumor** | | | | | | | |
| cT1 | Reference | | | | 1.00 | | |
| cT2 | 1.067 | 0.218 | 4.883 | <0.001 (<0.001) | 2.91 | 1.89 | 4.46 |
| cT3 | 2.079 | 0.243 | 8.546 | <0.001 (<0.001) | 8.00 | 4.96 | 12.88 |
| cT4 | 1.562 | 0.310 | 5.042 | <0.001 (<0.001) | 4.77 | 2.60 | 8.75 |
| **Sex** | | | | | | | |
| Male | Reference | | | | 1.00 | | |
| Female | 0.074 | 0.058 | 1.282 | 0.20 (0.99) | 1.08 | 0.96 | 1.21 |
| **Family history of retinoblastoma** | | | | | | | |
| Yes | Reference | | | | 1.00 | | |
| No | 0.322 | 0.175 | 1.839 | 0.07 (0.99) | 1.38 | 0.98 | 1.94 |
| **Hereditary retinoblastoma** | | | | | | | |
| Non-hereditary | Reference | | | | 1.0 | | |
| Hereditary[d] | -0.078 | 0.117 | -0.663 | 0.51 (0.99) | 0.93 | 0.74 | 1.16 |

[a]N=3267, number of events (i.e. enucleation) = 2177. 797 observations deleted due to missing observation time. Per patient, enucleation or exenteraion, primary or secondary, one or both eyes.

[b]Multiplied by 13, according to Bonferroni's model.

[c]Age included in the analysis as a continuous parameter. For further details on the relationship between age and log hazard for both survival and globe salvage, see "Relationship between age at time of diagnosis and log hazard" in the **Supplement**.

[d]Hereditary - bilateral/trilateral retinoblastoma, positive family history or positive blood *RB1* mutation (H1 in cTNMH).

Abbreviations: SE - Standard error, CI – confidence interval, mo – months, yr – years.

**eFigure 10. Competing risks for globe salvage: impute vs. delete cases with missing predictors**

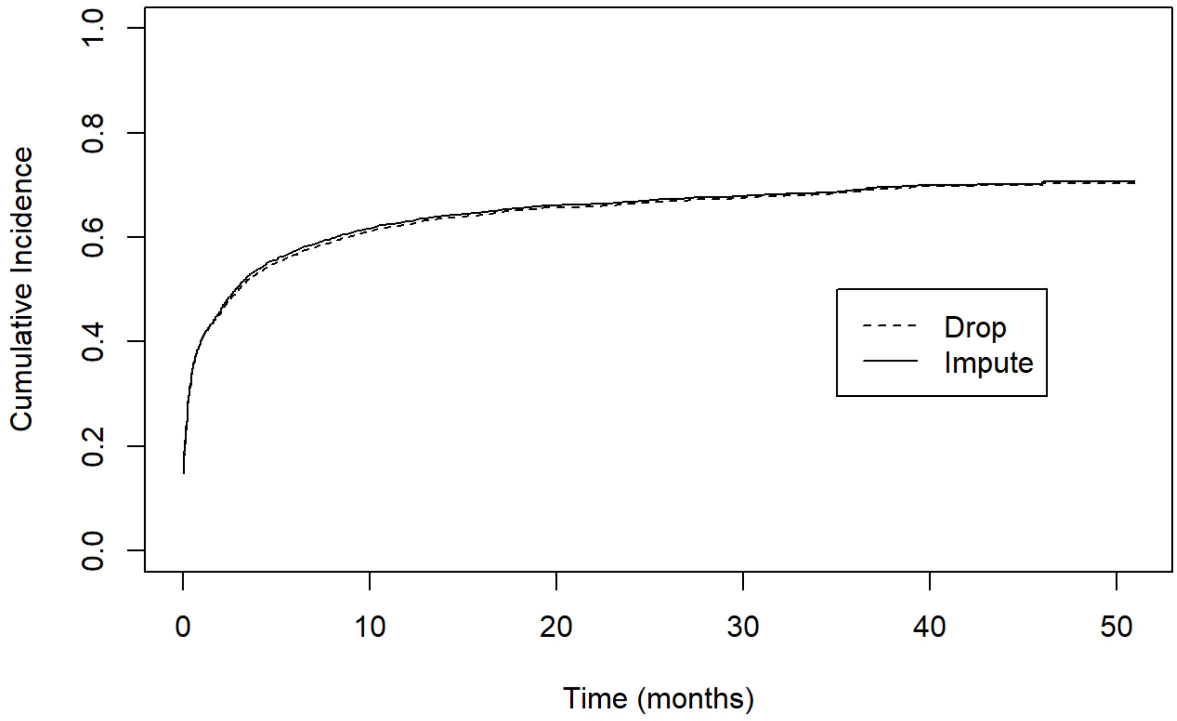


**eTable 12. Weighted Cox proportional hazard model for globe salvage in 4064 new patients with retinoblastoma diagnosed in 149 countries in 2017[a]: dropped missing x.**

| | Coefficient | Robust SE | Z value | P-value[b] | Hazard ratio | Lower 95% CI | Upper 95% CI |
|---|---|---|---|---|---|---|---|
| **Country of residence income** | | | | | | | |
| Low | Reference | | | | 1.00 | | |
| Lower-middle | -0.169 | 0.127 | -1.332 | 0.18 (0.99) | 0.84 | 0.66 | 1.08 |
| Upper-middle | -0.292 | 0.229 | -1.273 | 0.20 (0.99) | 0.75 | 0.48 | 1.17 |
| High | -0.130 | 0.173 | -0.749 | 0.45 (0.99) | 0.88 | 0.63 | 1.23 |
| **Age[c]** | | | | | | | |
| Age HR/mo | 0.007 | 0.002 | 3.108 | 0.002 (0.025) | 1.01 | 1.00 | 1.01 |
| Age>4yr HR/mo | -0.013 | 0.004 | -3.528 | <0.001 (0.005) | 0.99 | 0.98 | 0.99 |
| **Bilaterality** | | | | | | | |
| Unilateral | Reference | | | | 1.00 | | |
| Bilateral | -0.528 | 0.143 | -3.695 | <0.001 (0.003) | 0.59 | 0.45 | 0.78 |
| **Primary tumor** | | | | | | | |
| cT1 | Reference | | | | 1.00 | | |
| cT2 | 1.149 | 0.225 | 5.100 | <0.001 (<0.001) | 3.16 | 2.03 | 4.91 |
| cT3 | 2.239 | 0.246 | 9.105 | <0.001 (<0.001) | 9.38 | 5.79 | 15.19 |
| cT4 | 1.688 | 0.320 | 5.274 | <0.001 (<0.001) | 5.41 | 2.89 | 10.12 |
| **Sex** | | | | | | | |
| Male | Reference | | | | 1.00 | | |
| Female | 0.051 | 0.053 | 0.976 | 0.329 (0.99) | 1.05 | 0.95 | 1.17 |
| **Family history of retinoblastoma** | | | | | | | |
| Yes | Reference | | | | 1.00 | | |
| No | 0.212 | 0.154 | 1.376 | 0.17 (0.99) | 1.24 | 0.91 | 1.67 |
| **Hereditary retinoblastoma** | | | | | | | |
| Non-hereditary | Reference | | | | 1.00 | | |
| Hereditary[d] | -0.037 | 0.118 | -0.316 | 0.75 (0.99) | 0.96 | 0.76 | 1.21 |

[a]N=3051, number of events (i.e. enucleation) = 2027. 656 observations deleted due to missing observation time. Per patient, enucleation or exenteraion, primary or secondary, one or both eyes.
[b]P-values are presented before correction by Bonferroni's model.
[c]Age included in the analysis as a continuous parameter. For further details on the relationship between age and log hazard for both survival and globe salvage, see "Relationship between age at time of diagnosis and log hazard" in the **Supplement**.
[d]Hereditary - bilateral/trilateral retinoblastoma, positive family history or positive blood *RB1* mutation (H1 in cTNMH).
Abbreviations: SE - Standard error, CI – confidence interval, mo – months, yr – years.

## Survival - Multisample Imputation - Enucleation

**eTable 13. Multisample imputation for globe salvage in 4064 new patients with retinoblastoma diagnosed in 149 countries in 2017.**

| **Parameter** | | **Estimated coefficients** | **Standard errors** |
|---|---|---|---|
| **Age** | Age | 0.007 | 0.002 |
| | Age>4yr | -0.012 | 0.004 |
| **Country of residence income** | Lower-middle | -0.172 | 0.118 |
| | Upper-middle | -0.295 | 0.200 |
| | High | -0.183 | 0.172 |
| **Laterality** | Bilateral | -0.442 | 0.153 |
| **Primary tumor** | cT2 | 0.963 | 0.227 |
| | cT3 | 1.950 | 0.262 |
| | cT4 | 1.437 | 0.319 |
| **Sex** | Female | 0.068 | 0.058 |
| **Family history of retinoblastoma** | Yes | 0.268 | 0.171 |
| **Hereditary retinoblastoma** | Yes | -0.112 | 0.133 |
